# Polysynaptic inhibition between striatal cholinergic interneurons shapes their network activity patterns in a dopamine-dependent manner

**DOI:** 10.1038/s41467-020-18882-y

**Published:** 2020-10-09

**Authors:** Matthijs C. Dorst, Anna Tokarska, Ming Zhou, Kwang Lee, Stefanos Stagkourakis, Christian Broberger, Sotiris Masmanidis, Gilad Silberberg

**Affiliations:** 1 Department of Neuroscience, Karolinska Institutet, 17177 Stockholm, Sweden; 2 Department of Neurobiology, University of California, Los Angeles, Los Angeles, CA 90095 USA; 3 Division of Biology and Biological Engineering 156-29, Tianqiao and Chrissy Chen Institute for Neuroscience, California Institute of Technology, Pasadena, CA 91125 USA; 4 Department of Biochemistry and Biophysics, Stockholm University, Stockholm, 106 91 Sweden

**Keywords:** Neural circuits, Neurotransmitters

## Abstract

Striatal activity is dynamically modulated by acetylcholine and dopamine, both of which are essential for basal ganglia function. Synchronized pauses in the activity of striatal cholinergic interneurons (ChINs) are correlated with elevated activity of midbrain dopaminergic neurons, whereas synchronous firing of ChINs induces local release of dopamine. The mechanisms underlying ChIN synchronization and its interplay with dopamine release are not fully understood. Here we show that polysynaptic inhibition between ChINs is a robust network motif and instrumental in shaping the network activity of ChINs. Action potentials in ChINs evoke large inhibitory responses in multiple neighboring ChINs, strong enough to suppress their tonic activity. Using a combination of optogenetics and chemogenetics we show the involvement of striatal tyrosine hydroxylase-expressing interneurons in mediating this inhibition. Inhibition between ChINs is attenuated by dopaminergic midbrain afferents acting presynaptically on D2 receptors. Our results present a novel form of interaction between striatal dopamine and acetylcholine dynamics.

## Abstract (web summary)

The mechanisms underlying striatal cholinergic interneurons (ChINs) synchronization and its interaction with dopamine release are unclear. Here, the authors showed that polysynaptic inhibition between ChINs shapes their network activity and is mediated by dopaminergic input.

## Introduction

Striatal activity is shaped by acetylcholine, originating from striatal cholinergic interneurons (ChINs) and brainstem afferents^1^, and by dopamine (DA) from midbrain afferents. The dynamic interplay between striatal dopamine levels and ChIN activity is crucial for striatal function and underlies various reward, attention, and learning-related functions^2–8^. Early work suggested an antagonistic relationship in which elevated dopamine activity coincides with a decrease in the firing of ChINs^3,9–12^, yet more recent studies have shown an additional form of interaction, whereby synchronized ChIN activity promotes local dopamine release by acting on nicotine receptors of midbrain dopamine axons^1,13,14^. This indicates that dopamine release shapes striatal acetylcholine levels while also being reciprocally affected by them, specifically depending on the degree of synchronicity between ChINs. Synchronized ChIN activity can also inhibit medium spiny neurons (MSNs) by a feed-forward inhibitory pathway^15^, thus shaping striatal output to downstream basal ganglia nuclei. While the interplay between the cholinergic and dopaminergic systems in the striatum has important functional consequences, the underlying cellular and circuit mechanisms remain poorly understood.

The spiking activity of neighboring ChINs has been shown to be correlated^16–18^, and synchronized cessation of firing (pause) is associated with reward-related events^2,3,9,19^. These synchronized pauses are believed to be important for encoding salient sensory stimuli as well as learning of rewarding and aversive events^2,7–9,19,20^. In primates, synchronized pauses in neighboring ChINs exhibit different patterns with respect to the bursts preceding and following the pause. Some ChINs exhibit a reward-related burst-pause-rebound pattern while others have only a pause-rebound or an isolated pause response^2,10,19,21^. The mechanisms underlying the various pause responses are still unclear^22^. While the pause is modulated by dopamine from midbrain afferents^3,23–26^, dopamine alone does not explain the observed synchronicity in spiking activity. On the contrary, in dopamine-depleted animals, increased spike synchronization between ChINs was observed^16^ despite a decrease in synchronized pauses^3^. Such correlated activity could emerge due to common excitatory input from cortical and thalamic afferents^27,28^ as well as inhibitory afferents from the midbrain^29^ and globus pallidus^30–32^. In addition to these excitatory and inhibitory extrinsic sources, correlated ChIN activity may also be shaped by intrastriatal pathways^33–35^. The mechanisms underlying the synchronized activity of ChINs, specifically the different variants of bursts and pauses, are not known.

Here we propose that the activity patterns of striatal ChINs are shaped by a strong and widespread polysynaptic inhibition between them, which is attenuated by dopamine input acting via D2 receptors. Moreover, we identify and characterize two inhibitory afferent input pathways onto ChINs in the dorsal striatum from midbrain GABAergic and dopaminergic neurons. Our results suggest a novel form of interaction between the dopaminergic and cholinergic systems, in which dopamine release shapes striatal acetylcholine dynamics by acting directly on the recurrent inhibition between ChINs, thereby tuning their level of synchronous activity.

## Results

### Polysynaptic inhibition between ChINs

**Fig. 1:**
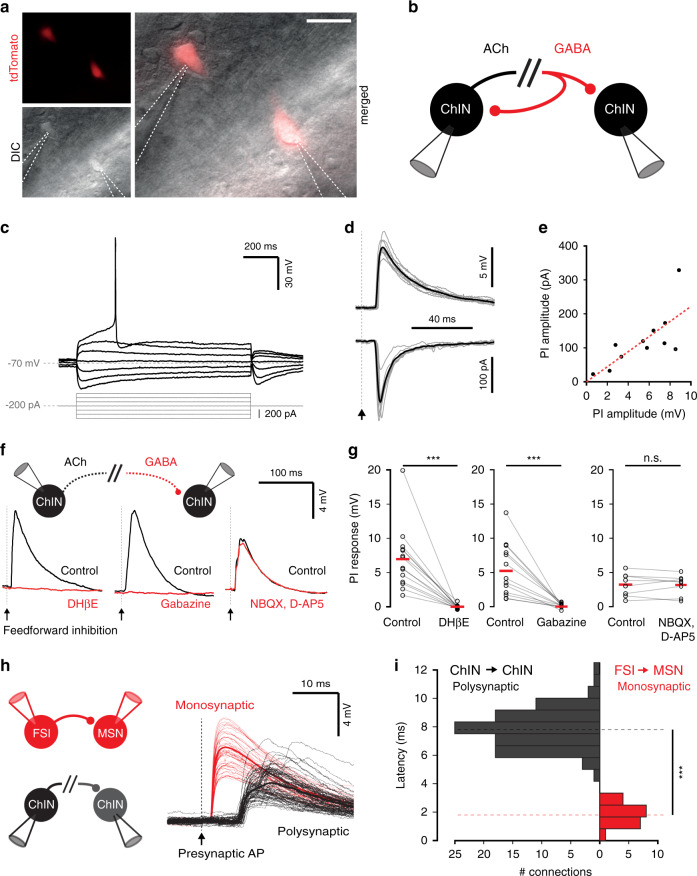
Cholinergic interneurons are connected by a strong polysynaptic inhibitory pathway. **a** Neurons expressing acetylcholine transferase (ChAT) are labeled in a ChAT-Cre mouse crossed to a tdTomato reporter and patched in whole-cell patch clamp configuration (*n* = 35 ChINs recorded in this configuration from 15 mice). Scale bar: 30 µm. **b** Schematic representation of a paired whole-cell recording from two neighboring ChINs expressing feedback and feed-forward polysynaptic inhibition. **c** ChINs are identified by their typical I–V response, including a pronounced sag, wide action potentials, depolarized membrane potential, and moderate inward rectification. **d**, **e** Example of a feed-forward inhibitory connection between ChINs as recorded in current- and voltage-clamp configurations. The synaptic currents scaled according to the voltage responses by 22.2 ± 2.9 pA mV^−1^ (two-sided linear regression through the origin, *R*^2^ = 0.86, *n* = 11 connections, *p* = 0.000016). **f**, **g** Polysynaptic inhibition was blocked by bath application of the nicotinic receptor antagonist DHβE (*n* = 14 connections, *Z* = −3.296, *p* = 0.00066) and the GABA_A_ receptor antagonist gabazine (*n* = 14 connections, *Z* = −3.296, *p* = 0.00098) but unaffected by AMPA and NMDA receptor antagonists NBQX and D-AP5 (*n* = 8 connections, *Z* = −0.14, *p* = 0.89, 2-tailed Wilcoxon signed-ranks test). **h** Example of a monosynaptic inhibitory connection from a fast-spiking interneuron (FSI) onto an MSN and a polysynaptic feed-forward inhibition between ChINs. Compared to the monosynaptic FSI-MSN connection, polysynaptic ChIN-ChIN connections exhibit longer onset latencies and trial to trial variability. **i** Onset latency histogram for monosynaptic connections between FSIs and MSNs (in red), compared to the polysynaptic connection between ChINs (in black). Mean latency for the polysynaptic connection (7.8 ms, *n* = 98 connections) is significantly larger (*p* = 3.0E−43, two-sided independent samples *t*-test) than the monosynaptic FSI onto MSN connection (1.8 ms, *n* = 20 connections).

**Fig. 2:**
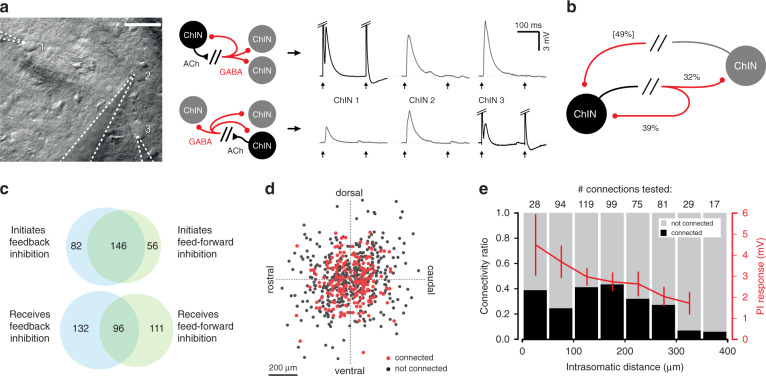
Polysynaptic inhibition between ChINs is a ubiquitous intrastriatal inhibitory pathway. **a** In multi-neuron patch clamp configuration polysynaptic responses can be observed in multiple postsynaptic ChINs simultaneously (*n* = 30 cases of divergent connectivity), elicited by neighboring presynaptic ChINs (gray traces), as well as feedback inhibition onto the same ChIN (black traces, *n* = 846 observed examples). Scale bar: 40 µm. **b** Probability of feed-forward inhibition (32%) is slightly lower than feedback inhibition (39%). In pairs of ChINs exhibiting feed-forward inhibition, reciprocal connectivity probability increases to 49%. **c** Venn diagrams showing the probabilities for recording feedback and feed-forward polysynaptic inhibition between ChINs. The majority of ChINs (64%) initiating feed-forward inhibition also initiate feedback inhibition, though ChINs receiving feed-forward inhibition exhibit only marginally more feedback inhibition (42% versus 37%). **d** Relative spatial distribution of 620 tested pairs. Polysynaptic inhibition does not exhibit a significant directional preference within the dorso-ventral and rostro-caudal axes in parasagittal slices. **e** Connection probability (black/gray bars) ranges from 24 to 43% for 496 potential connections tested within 300 µm (numbers above bars denote tested connections per bin). Feed-forward response amplitude (red trace, mean amplitude per bin ± s.e.m.) shows a weak distance-dependent decrease (r_S_(183) = −0.15, *p* = 0.048).

Pairs, triplets and quadruples of ChINs were recorded in whole-cell patch clamp mode in the mouse dorsal striatum and polysynaptic inhibitory connections between them were studied. ChINs were identified by their large somata and typical electrophysiological properties, such as a depolarized membrane potential and I_h_-mediated sag response (Fig. 1a–c). In some experiments, ChINs were identified by fluorescent labeling using ChAT-Cre mice crossed with tdTomato expressing reporter mice (Fig. 1a). Inhibitory polysynaptic responses were evoked in 32% (*n* = 202/629 tested pairs) of ChINs following stimulation of a neighboring presynaptic ChIN with a single action potential (Fig. 1d). In 39% of cells (*n* = 297/769 ChINs), an action potential in a presynaptic ChIN induced a feedback inhibitory response on itself (Figs. 1b, 2a). We used a high-chloride concentration in the patch-pipette, resulting in depolarizing responses that enabled us to clearly observe both feed-forward and feedback GABAergic responses (Supplementary Fig. 1). Under these conditions the amplitude of synaptic responses triggered by a single presynaptic action potential was 3.4 ± 0.2 mV (*n* = 202 connections) for feed-forward connections and 4.7 ± 0.2 mV (*n* = 297 connections) for feedback connections, with some connections being larger than 15 mV in amplitude. In a subset of pairs, polysynaptic responses were recorded in both current- and voltage-clamp, enabling the extraction of a linear regression of 22 ± 3 pA mV^−1^ (*R*^2^ = 0.86, *n* = 11 connections) and peak synaptic conductance of 3.4 ± 0.7 pS (ranging between 0.9 and 9.4 pS) for feed-forward polysynaptic connections (Fig. 1d, e). As reported previously^35^, these polysynaptic responses were blocked by DHβE, an antagonist for nicotinic receptors composed of α4β2 subunits, and by the GABA_a_ receptor antagonist SR-95531 (gabazine), but were unaffected by the glutamate receptor antagonists NBQX and D-AP5 (Fig. 1f, g). The onset of responses was 7.8 ± 0.1 ms (ranging between 5.4 and 10.6 ms, *n* = 98 connections) and they were often compound responses, containing multiple synaptic events. In contrast, monosynaptic connections such as those between striatal fast-spiking interneurons and MSNs recorded under the same conditions, had shorter latencies (1.8 ± 0.1 ms, *n* = 20 pairs) and narrower onset distributions (Fig. 1h, i).

### Network properties of polysynaptic inhibition

Polysynaptic connections between ChINs were common and exhibited a high degree of divergence and convergence, such that in multineuron patch clamp recordings a presynaptic ChIN inhibited several neighboring ChINs and postsynaptic ChINs received inhibition from multiple presynaptic ChINs (Fig. 2a). In simultaneous recordings, we observed reciprocal, divergent, and convergent connections, including triplets and quadruplets displaying all-to-all connectivity in which each ChIN inhibited all of its neighbors as well as itself. Paired-pulse stimulation at 200 ms intervals demonstrated almost complete depression of the synaptic response, as shown previously^35^. For ChINs evoking feed-forward inhibition onto their neighbors (32% of pairs), we found a higher probability (49%, *n* = 92/192) for receiving reciprocal inhibition from their targets (Fig. 2b). Likewise, ChINs initiating feedback inhibition onto themselves were more likely to receive input from other ChINs (42%, 96/228 ChINs) and produce feed-forward inhibition (64%, 146/228 ChINs, Fig. 2c). Polysynaptic responses were recorded between ChINs with intersomatic distances of 16–520 µm (mean distance 165.4 ± 5.9 µm) with no apparent spatial preference within the dorso-ventral and rostro-caudal axes in sagittal slices (Fig. 2d). Within the range of intersomatic distances of 300 µm, the strength and probability of feed-forward connectivity remained stable, with connections beyond that range found less often (*p* < 0.000002, *Z* = −4.851, 2-tailed Mann–Whitney *U* test, *n* = 629 potential feed-forward connections) (Fig. 2e and Supplementary Fig. 2), likely to be affected also by the topological limitations of the slice preparation. These results show that polysynaptic inhibition between ChINs is a prevalent and strong inhibitory pathway linking neighboring ChINs within the striatal microcircuitry.

### Single ChINs can broadcast simultaneous pauses in postsynaptic ChINs

**Fig. 3:**
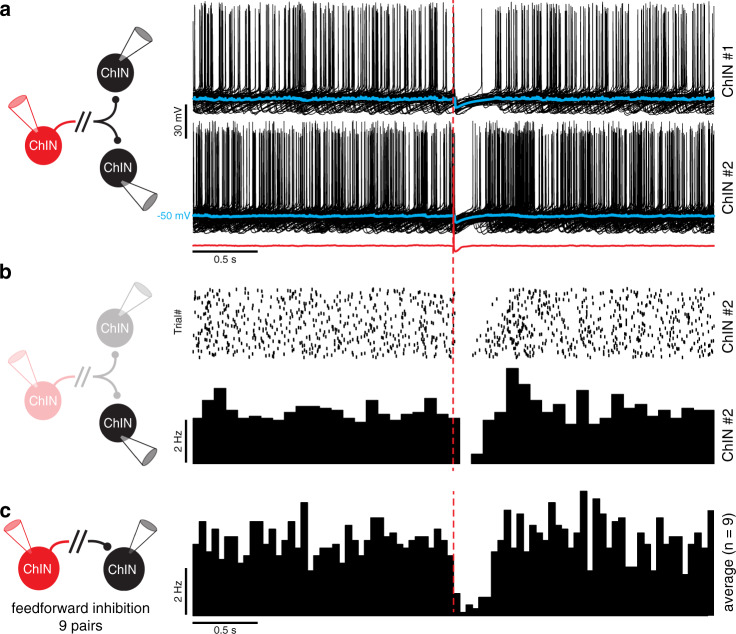
Synchronized pauses in ChINs mediated by polysynaptic inhibition. **a** A single action potential in a presynaptic ChIN (in red) inhibits neighboring ChINs, inducing simultaneous pauses. Postsynaptic sweeps (in black) show a clear pause in firing in postsynaptic neurons and rebound firing after the pause in ChIN #2. The average traces are overlaid in blue. The chloride reversal potential is ~ −70 mV, resulting in hyperpolarizing GABA-mediated response. **b** The corresponding raster plot of ~200 repetitions and spike time histogram of ChIN #2. **c** Spike time histogram of 9 connected ChIN pairs recorded in the same configuration described above. Note the pause in the postsynaptic firing of ~300 ms following the presynaptic action potential (time designated in red dashed line).

In order to test the impact of polysynaptic inhibition on ChIN activity, we stimulated individual presynaptic ChINs while simultaneously recording the spontaneous firing in postsynaptic ChINs (Fig. 3a). In these experiments, we used a low-chloride intracellular solution to obtain physiological inhibitory responses (reversal potential of roughly −70 mV) for GABAa inputs. Single presynaptic APs induced pronounced pauses in postsynaptic ChINs, often followed by an increase in AP discharge frequency (Fig. 3b, c). This suggests that a single AP in a presynaptic ChIN can induce synchronous activity patterns in its postsynaptic targets by polysynaptic IPSPs. Indeed, using triple patch clamp recordings we could induce simultaneous polysynaptic responses in two target ChINs by a single presynaptic action potential, causing synchronized pauses in both (Fig. 3a, b). These results show that the robust inhibition between spontaneously active ChINs can act as a mechanism for synchronizing their activity and pause patterns via an intrastriatal mechanism. Specifically, simultaneous spiking in presynaptic ChINs can efficiently broadcast pauses to multiple neighboring ChINs.

### Network activity of TANs is shaped by intrastriatal inhibition

**Fig. 4:**
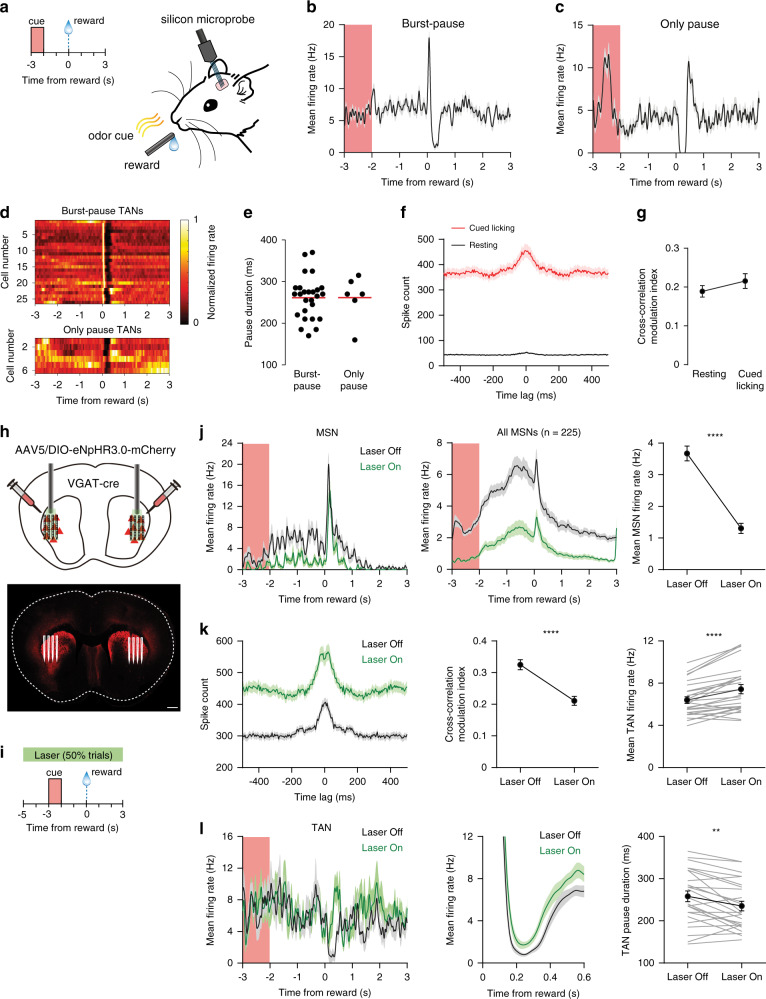
ChINs exhibit synchronous activity and GABAergic interneuron-dependent pauses in vivo. **a** Silicon microprobe recordings in vivo show TAN activity during a cue reward task. **b** Mean firing rate versus time of a TAN exhibiting a burst-pause response to rewards. **c** Mean firing rate versus time of a TAN exhibiting an only pause response to rewards. **d** Mean normalized firing rate versus time of all 26 reward burst-pause TANs (top) and 6 only pause TANs (bottom). **e** There is no significant difference in the mean pause duration of burst-pause and only pause TANs (*n* = 26 burst-pause and 6 only pause TANs, two-sided unpaired *t*-test, *p* = 0.99). **f** Mean spike time cross-correlogram of 65 simultaneously recorded pausing TAN pairs. Black and red curves represent data from the entire recording session, and a 300 s resting period, respectively. **g** There is no significant difference in the cross-correlation modulation index between spiking activity during resting and cued licking (*n* = 65 pausing TAN pairs, two-sided paired *t*-test, *p* = 0.2). **h** Top: schematic of viral injections of eNpHR3.0 in the lateral striatum of VGAT-Cre mice. Injections were carried out bilaterally, allowing recording in both hemispheres (one hemisphere per recording session). Bottom: confocal image of eNpHR3.0 expression (red) and the approximate position of the inserted silicon microprobe (white lines). Scale bar: 0.5 mm. **i** Schematic showing the timing of the laser stimulus used to optogenetically inhibit GABAergic cells during the Pavlovian reward conditioning task. The laser was applied on 50% of randomly selected trials. **j** Mean firing rate versus time of one MSN (left) and all 225 recorded MSNs (middle) during Laser Off (black) and Laser On (green) trials. Right: MSN activity was significantly reduced during Laser On trials (*n* = 225 MSNs, two-sided paired *t*-test, *p* = 7.6E−27). **k** Mean spike time cross-correlogram of 87 simultaneously recorded pausing TAN pairs (left). Black and green lines represent data from Laser Off and Laser On trials, respectively. Middle: the cross-correlation modulation index was significantly lower during Laser On trials (*n* = 87 TAN pairs, two-sided paired *t*-test, *p* = 3.6E−24). Right: TAN activity was significantly increased during Laser On trials (*n* = 26 TANs, two-sided paired *t*-test, *p* = 0.000007). **l** Mean firing rate versus time of one TAN (left) and all pausing TANs during the reward pause period (middle). Right: the mean pause duration was significantly reduced during Laser On trials (*n* = 25 TANs, two-sided paired *t*-test, *p* = 0.002). All data represent mean ± SEM.

Since the activity patterns of striatal ChINs are believed to play an important role in behavior^36,37^, we used in vivo electrophysiological recordings to examine whether the synchronized activity and different modes of reward-related pauses can also be observed in awake behaving mice. Head-fixed mice (*n* = 10 mice) were trained on a Pavlovian reward conditioning task, in which olfactory cues were paired with an unconditioned sweetened milk reward (See “Methods”, Fig. 4a). After repeated cue-reward pairings, animals developed an anticipatory licking response that preceded reward delivery. Recordings were performed in well-trained animals using silicon microprobes^38^, and cells were classified as tonically active neurons (TANs, putative ChINs^39^), MSNs, or FS interneurons, based on their spike waveform and the rate and regularity of their baseline firing (see “Methods” and Supplementary Fig. 3a)^40^.

A significant fraction of TANs (59%, 32/54) displayed a brief pause in firing after reward delivery (Fig. 4b–e), whereas this fraction was lower for the MSN and FSI populations (Supplementary Fig. 3b). There was some variability in TAN activity that preceded the pause. To examine this further, we subdivided TANs into two populations, corresponding to cells with a burst preceding the pause (burst-pause, *n* = 26/54 TANs, Fig. 4d, top) and those without any initial burst (only-pause, *n* = 6/54 TANs, Fig. 4d, bottom). In addition to the burst preceding the pause, a rebound burst following the pause was observed in some cells (*n* = 12/26 burst-pause and 3/6 only-pause TANs). Furthermore, the duration of the pause was similar between the burst-pause (mean ± SEM: 261 ± 10 ms) and the only-pause (262 ± 22 ms) populations (Fig. 4e), suggesting a highly coordinated circuit mechanism for producing a pause across the striatal TAN network. To find evidence for such coordinated activity, we examined spike time cross-correlations between pairs of simultaneously recorded TANs (*n* = 188 TANs, 65 pairs). TANs tended to fire synchronously on a time scale of about 100 ms, both during periods corresponding to the behavioral task and resting (Fig. 4f, g). The synchrony, as measured by the cross-correlation modulation index (see Methods), was more pronounced for neighboring cell pairs and decreased with greater pairwise distance (Supplementary Fig. 3c).

To study the involvement of striatal GABAergic neurons in shaping TAN activity we expressed a cre-dependent inhibitory opsin (eNpHR3.0) in the dorsolateral striatum of VGAT-Cre mice. After training animals on the Pavlovian reward conditioning task, we recorded in vivo striatal activity while optogenetically inhibiting VGAT-positive cells (Fig. 4h). During the measurement, 50% of randomly selected trials were paired with a continuous laser stimulus (8 s duration, Fig. 4i). While this unilateral optogenetic manipulation had no significant effect on anticipatory licking behavior (*n* = 7 recording sessions with laser stimulation, Supplementary Fig. 3d), it significantly reduced the mean MSN firing rate (*n* = 225 MSNs, Fig. 4j). In contrast, TANs with reward pause responses showed higher mean firing rates (*n* = 26 TANs, Fig. 4k). Despite the increase in TAN firing rate, the cross-correlation modulation index from simultaneously recorded TAN pairs (*n* = 87 pairs) was significantly reduced by the laser (Fig. 4k), suggesting a desynchronization of TAN network activity. The light-induced increase in TAN activity was also accompanied by a reduction in the pause duration (Fig. 4l). Taken together, these results demonstrate that, as shown previously in primates^10,16^ and rats^28^, TANs in the mouse striatum display synchronized activity and a robust pause response to rewarding events, which is often, but not always, preceded by a burst in activity. Moreover, the increase in TAN firing frequency, the decrease in firing synchrony and shortening of the pause following optogenetic inhibition of striatal GABAergic neurons, supports the involvement of intrastriatal inhibition in shaping the network activity of TANs.

### TH interneurons mediate inhibition between ChINs

**Fig. 5:**
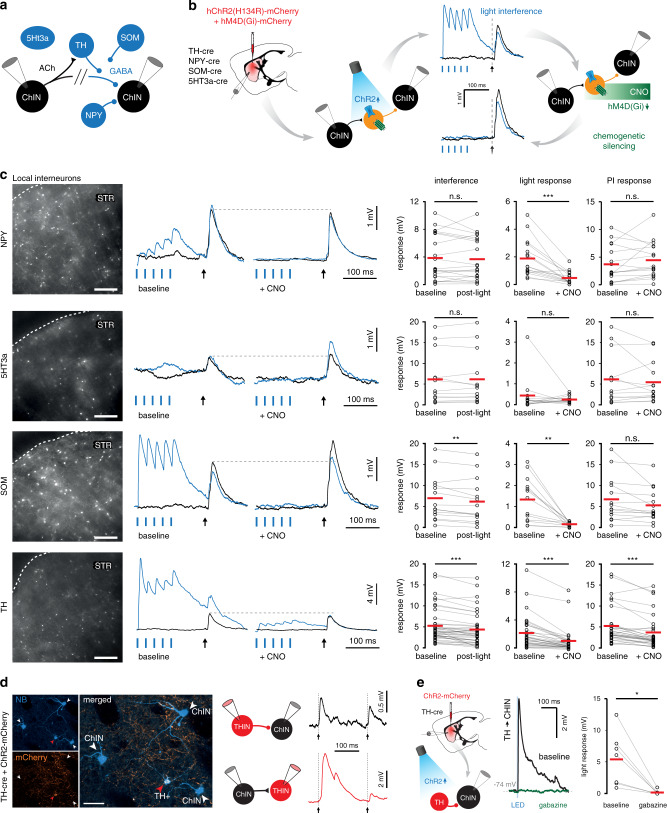
Assessment of the involvement of striatal interneurons in mediating polysynaptic inhibition. **a** Multichannel recordings suggest TH-expressing interneurons partially mediate the polysynaptic inhibition between ChINs, with further modulation by SOM-expressing interneurons. **b** Various transgenic mouse lines were used to selectively express hChR2(H134R)-mCherry and hM4D(Gi)-mCherry in striatal interneurons. Dual expression of an excitatory opsin and inhibitory DREADD enables full control and verification of the effectiveness of inhibition in each population: light-induced interference suggests involvement in polysynaptic inhibition. Subsequent chemogenetic silencing further validates this involvement. Sagittal brain schematic adapted from the Allen Institute for Brain Science, Allen Mouse Brain Atlas. Available from: https://mouse.brain-map.org/static/atlas. **c** Polysynaptic responses were recorded under baseline conditions, following a 40 Hz train of light pulses and in the presence of CNO. Light-induced activation reduced the amplitude of polysynaptic inhibition for SOM- and TH-expressing interneurons (*n* = 14 connections, *p* = 0.006 and *n* = 40 connections, *p* = 0.000012, respectively, two-tailed Wilcoxon signed-ranks test). Light-induced input on striatal ChINs was reduced in all cases following bath application of CNO. Polysynaptic inhibition was reduced following bath application of CNO in TH-expressing interneurons (*n* = 31 connections, *p* = 0.000036, two-tailed Wilcoxon signed-ranks test). Scale bars: 250 µm. **d** TH-expressing interneurons transduced with hChR2(H134R)-mCherry were recorded and filled with neurobiotin (NB) together with nearby ChINs to assess direct connections. ChINs receive direct synaptic input from TH neurons (top, *n* = 13 TH to ChIN connections) and vice versa (bottom, *n* = 11 ChIN to TH connections). Note strong desensitization of the nicotinic receptor on repeated stimulation. Scale bar: 50 µm. **e** Light-induced input from THINS onto ChINs was abolished in the presence of gabazine (*n* = 7 postsynaptic responses, *Z* = −2.366, *p* = 0.02 two-sided Wilcoxon signed-ranks test).

We next wanted to determine which type of striatal interneuron is involved in mediating polysynaptic inhibition between ChINs. Striatal ChINs receive inhibitory input from various striatal interneurons such as the neuropeptide-Y (NPY) interneurons^15^, 5-hydroxytryptamine 3a receptor (5HT3a) expressing interneurons^41,42^, somatostatin (SOM) interneurons^43,44^, and provide excitatory input to tyrosine hydroxylase-expressing interneurons (THINs)^44,46^. In contrast, parvalbumin (PV) expressing interneurons are only sparsely interconnected with ChINs^15,47^. In order to elucidate which interneuron type mediates polysynaptic inhibition between ChINs, we employed a dual-transduction viral approach whereby specific interneuron types expressed both the excitatory opsin ChR2 and the inhibitory DREADD receptor hM4D(Gi) in a cre-dependent manner (Fig. 5a, b). We quantified the attenuation in polysynaptic inhibition between neighboring ChINs following an optogenetic interference protocol (5 pulses at 40 Hz). Modulation of the polysynaptic inhibition following optogenetic interference would indicate that the transduced interneuron population is involved in the polysynaptic pathway. Repeating this protocol in the presence of bath-applied CNO provided further support to the role of the selected interneuron population in mediating polysynaptic inhibition (Fig. 5a–c). This experimental configuration also enabled us to monitor the efficiency of chemogenetic silencing of the targeted population and compare it to the effect on polysynaptic responses. No effect of optogenetic interference was observed in interneurons expressing NPY or 5HT3a, with the latter population only weakly inhibiting striatal ChINs (Fig. 5c). Modulation of polysynaptic inhibition was observed following optogenetic interference in both SOM-expressing interneurons and THINs. As expected, chemogenetic silencing strongly reduced all light-evoked responses from NPY (*p* < 0.001, *Z* = −3.464, *n* = 16 ChINs, 2-tailed Wilcoxon signed-ranks test), SOM (*p* = 0.001, *Z* = −3.180, *n* = 13 ChINs) and TH (*p* < 0.001, *Z* = −4.840, *n* = 31 ChINs) expressing interneurons; however, polysynaptic inhibition between ChINs was exclusively reduced by chemogenetic silencing of THINs (*p* < 0.001, *Z* = −4.135, *n* = 31 connections) (Fig. 5c). These results show that from all the tested interneuron types, only THINs are involved in mediating the polysynaptic pathway between ChINs. We further studied the direct synaptic connectivity between THINs and ChINs by obtaining simultaneous whole-cell recordings from neighboring neurons of both types (Fig. 5d), and also by focal optogenetic activation of THINs (Supplementary Fig. 4a). In 13/50 tested connections, action potentials in THINs evoked synaptic responses in ChINs and a similar connection probability (11/41 tested pairs) was seen for direct connections from ChINs onto THINs (Fig. 5d and Supplementary Fig. 4a). Light-evoked responses from THINs onto ChINs were abolished by bath application of gabazine (*p* = 0.018, *Z* = −2.366, *n* = 7, Wilcoxon Signed-ranks test, Fig. 5e) whereas nicotine puffs induced action potential discharge in THINs recorded in cell-attached mode (Supplementary Fig. 4b–e). The responses of THINs to nicotine puffs were unaffected by glutamate receptor antagonists (Supplementary Fig. 4b–h). These results show a synaptic substrate for polysynaptic inhibition between ChINs mediated by THINs via nicotinic and GABAergic synapses.

### Striatal ChINs receive inhibitory input from midbrain neurons

**Fig. 6:**
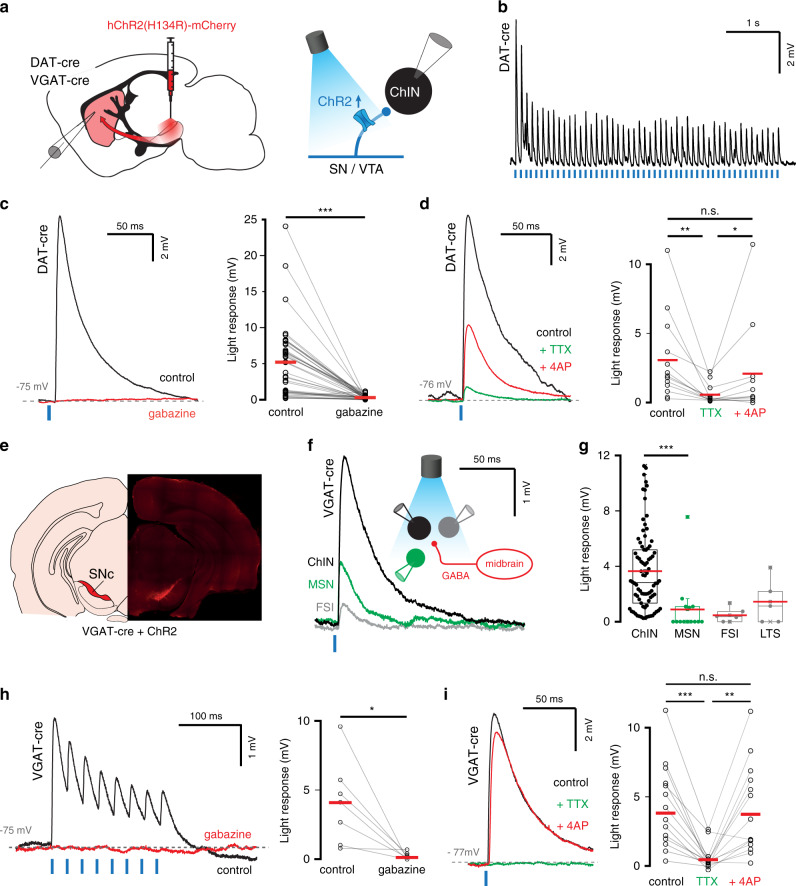
Cholinergic interneurons in dorsal striatum receive input from midbrain dopaminergic and GABAergic neurons. **a** Neurons in the midbrain of DAT-Cre and VGAT-Cre mice were transduced with ChR2-mCherry and polysynaptic responses were recorded in striatal ChINs following light evoked activation of midbrain afferents. **b** In DAT-Cre mice, repeated activation of afferent terminals reliably induced light-evoked responses in ChINs, which **c** were abolished in the presence of gabazine (*n* = 38 ChINs, *p* = 1.5E−7, two-tailed Wilcoxon signed-ranks test). **d** Light-induced responses were reduced by TTX (*n* = 13 striatal neurons exhibiting light-evoked responses, *Z* = −3.180, *p* = 0.0015 two-tailed Wilcoxon signed-ranks test) but recovered following subsequent application of 4-AP (*n* = 11 neurons, *Z* = 2.293, *p* = 0.022 two-tailed Wilcoxon signed-ranks test) to amplitudes comparable to baseline (*n* = 11 neurons, *Z* = −1.867, *p* = 0.062, two-tailed Wilcoxon signed-ranks test) indicating monosynaptic input. **e** Location of transduced neurons in a VGAT-Cre mouse. **f** Example of a simultaneous whole-cell recording of neighboring MSN, ChIN, and FSI, all responding to light-evoked activation of GABAergic terminals of VGAT-Cre midbrain neurons. **g** ChINs receive stronger GABAergic input from midbrain projections than MSNs (*n* = 82 & 15 cells, *p* = 0.000004, two-tailed Mann–Whitney *U* test). GABAergic interneurons (low threshold spiking—LTS, and fast-spiking interneurons—FSI) respond weakly to optogenetic stimulation of midbrain GABAergic afferents. Boxplots indicate minima/maxima (whiskers), first/third quartile (box), median (black bar) and mean (red line). **h** Light-evoked responses are abolished in the presence of 10 µM gabazine (*n* = 8 ChINs, *p* = 0.011, two-tailed paired *t*-test). **i** Light-induced responses were reduced by TTX (*n* = 13 ChINs exhibiting light-evoked responses, *Z* = −3.180, *p* = 0.0015 two-tailed Wilcoxon signed-ranks test) but recovered following subsequent application of 4-AP (*n* = 12 ChINs, *Z* = 2.981, *p* = 0.0029 two-tailed Wilcoxon signed-ranks test) to amplitudes comparable to baseline (*n* = 12 ChINs, *Z* = −0.392, *p* = 0.70, two-tailed Wilcoxon signed-ranks test) indicating monosynaptic input.

Striatal ChINs also receive inhibitory input from the midbrain, mediated by both GABAergic and dopaminergic neurons^23,26,31,37,48^. Furthermore, dopaminergic terminals were suggested to mediate ChIN-driven inhibition of MSNs^48^. In this scenario, nicotinic receptors on dopaminergic terminals are activated by synchronous firing of ChINs, resulting in dopamine release^14^ as well as co-release of GABA^49^. We, therefore, investigated whether dopaminergic axons from the midbrain provide inhibitory input to ChINs in the dorsal striatum and whether this pathway is involved in mediating the polysynaptic inhibition. To that end, we used DAT-Cre mice^50^ to express ChR2 in dopaminergic midbrain neurons by viral injection (Fig. 6a and Supplementary Figs. 5a, 6a–d). Optogenetic stimulation in the dorsal striatum resulted in synaptic responses in ChINs that were blocked by bath application of 10 µM gabazine (94 ± 1.5% attenuation, *n* = 49 neurons, Fig. 6c). These GABAergic responses were monosynaptic, as they had short onset latencies (2.4 ± 0.1 ms) and persisted after application of tetrodotoxin (TTX, 0.5 µM) and 4-aminopyridine (4-AP, 100 µM) (Fig. 6d). Synaptic responses were robust and evident even following long stimulation trains (Fig. 6b) showing that GABA release is sustained during the ongoing activity of midbrain axons.

ChINs in the ventral striatum were shown to receive selective inhibitory input from midbrain GABAergic neurons^29^. We next investigated whether a similar pathway exists in dorsal striatum, and if it is involved in mediating polysynaptic inhibition between ChINs. To that end, we virally expressed ChR2 in midbrain neurons of VGAT-Cre mice^51^ (Fig. 6a, e, and Supplementary Fig. 5d, e, see “Methods”). Retrograde labeling of these inputs showed that the majority of presynaptic neurons originate in the Substantia Nigra and to a lesser extent in the lateral Ventral Tegmental Area (Supplementary Fig. 5d, *n* = 2 mice). Photostimulation evoked synaptic responses in ChINs in dorsal striatum with short latency (2.3 ± 0.2 ms), which persisted after application of TTX (0.5 µM) and 4-AP (100 µM), thus indicating a monosynaptic pathway^52^ (Fig. 6i). As described for the nucleus accumbens^29^, light-evoked inhibitory inputs were strongest in ChINs in comparison to simultaneously recorded MSNs and GABAergic interneurons (Fig. 6f, g). Light-evoked responses were completely blocked by bath application of 10 µM gabazine, and optogenetic stimulation with light trains showed depressing synaptic responses (Fig. 6h).

### Midbrain afferents do not mediate inhibition between ChINs

**Fig. 7:**
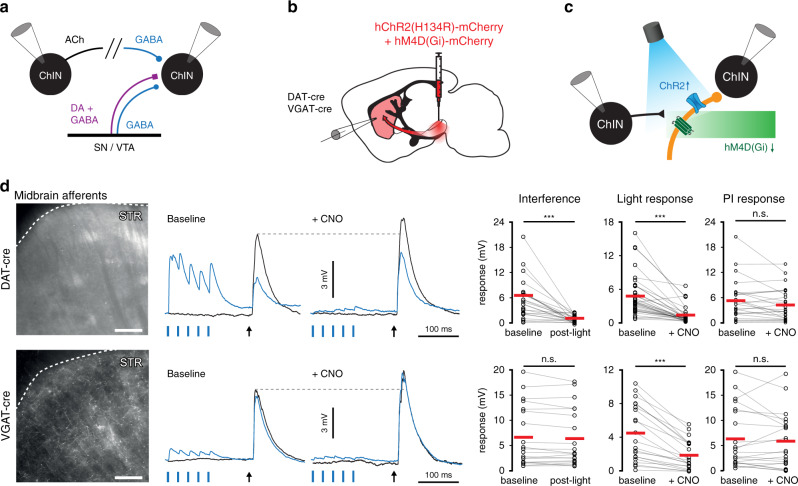
Activation of midbrain dopaminergic afferents induces monosynaptic GABA_A_ responses in Striatal ChINs and reduces polysynaptic inhibition. **a**–**c** Midbrain neurons in a VGAT-Cre or DAT-Cre mouse were virally transduced with hChR2(H134R)-mCherry and hM4D(Gi)-mCherry. Light-evoked responses were recorded in Striatal ChINs under baseline conditions and in the presence of CNO. **d** Polysynaptic responses were recorded under baseline conditions, following a 40 Hz train of light pulses and in the presence of CNO. No interference was detected in VGAT-Cre mice and polysynaptic inhibition was unaffected by CNO. In DAT-Cre mice, polysynaptic responses were strongly attenuated following light activation (*n* = 25 connections, *p* = 0.000036, two-tailed Wilcoxon signed-ranks test), but were unaffected by CNO (*n* = 26 connections, *p* = 0.12, two-tailed Wilcoxon signed-ranks test). Scale bars: 250 µm.

To test the participation of GABAergic and dopaminergic midbrain afferents in mediating polysynaptic inhibition between ChINs we used the same approach described above, in which photostimulation and chemogenetic silencing were used in VGAT-Cre and DAT-Cre mice (Fig. 7a–c). In both mouse lines chemogenetic silencing abolished light-evoked responses but did not attenuate polysynaptic inhibition between ChINs, suggesting that neither afferent pathway mediated it (Fig. 7d). However, photostimulation of dopamine terminals in the DAT-Cre mouse did attenuate polysynaptic inhibition while stimulation of VGAT-Cre midbrain terminals did not have any impact (Fig. 7d). These results suggest that dopamine released from DAT-Cre terminals could modulate the polysynaptic interactions between ChINs. In a subset of experiments, we transduced midbrain dopaminergic cells by injections of a retrograde AAV (pAAVrg-EF1a-double floxed-hChR2(H134R)-mCherry-WPRE-HGHpA) in the dorsal striatum of DAT-Cre mice, thus expressing ChR2 only in dopamine cells projecting to the dorsal striatum (Supplementary Fig. 5a). Also in these recordings, striatal photostimulation induced GABAergic responses in ChINs (Supplementary Fig. 5b) and optogenetic pre-pulses attenuated polysynaptic inhibitory responses (Supplementary Fig. 5c). We further wanted to verify that the GABAergic responses and the attenuation were not specific to the DAT-Cre mouse line. To that end we repeated the above experiments with TH-Cre mice^53^, in which we virally transduced ChR2 in midbrain dopamine cells. As in DAT-Cre mice, optogenetic stimulation of striatal terminals from midbrain evoked large responses that were fully blocked by gabazine and optogenetic pre-pulses attenuated polysynaptic responses by 80% (Supplementary Fig. 6b, c).

In order to further assess the contribution of dopamine terminals to polysynaptic inhibition between ChINs, we manipulated dopamine transmission through unilateral 6-OHDA lesions in the medial forebrain^54^ (Supplementary Fig. 7a–f) and by selective silencing of midbrain DAT-expressing neurons using Cre-dependent expression of Diphtheria Toxin-A (DTA) or Tetanus Toxin Light Chain (TeLC)^55^ (Supplementary Fig. 7g–j). The proportion of ChINs expressing polysynaptic inhibition was unaffected when midbrain dopamine neurons were lesioned with either 6-OHDA (*n* = 20/23 ChINs), DTA (*n* = 7/9 ChINs), or silenced by TeLC (*n* = 9/11 ChINs) compared to control (*n* = 10/12 ChINs). Our data show that polysynaptic inhibition between ChINs is not mediated by GABA release from midbrain terminals, but suggest instead that it is regulated by the release of dopamine.

### Inhibition between ChINs is attenuated by dopamine

**Fig. 8:**
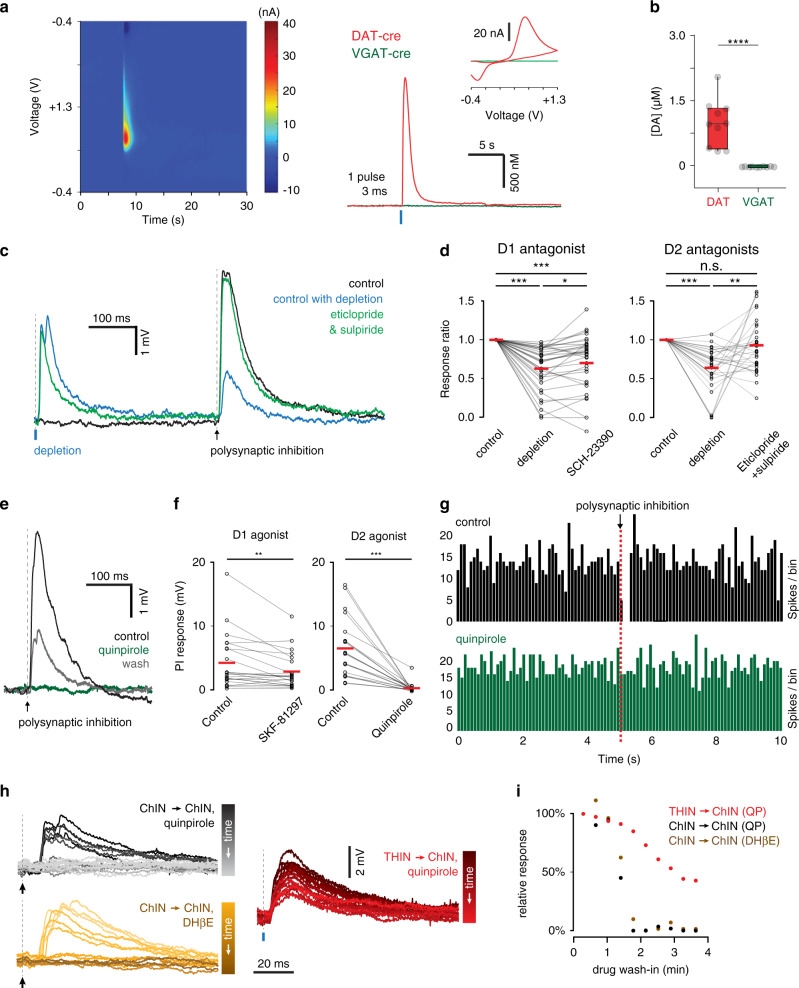
Dopamine induced interference of polysynaptic inhibition is mediated by D2 receptors. **a**, **b** Fast scan cyclic voltammetry measurements indicate light-evoked dopamine release in DAT-Cre, but not VGAT-Cre animals (*n* = 10 recording sites in each condition, *p* = 0.000091, two-sided Kolmogorov–Smirnov test). Boxplots indicate minimum, first quartile, median, third quartile, and maximum. **c** Example of polysynaptic responses exhibiting light-induced interference, which is abolished in the presence of D2 antagonists eticlopride and sulpiride. **d** Interference is reduced when light evoked dopamine release is combined with bath application of D1 receptor antagonist SCH-23390 from 39% to 30% (*n* = 29 connections, *p* = 0.013, two-tailed paired samples *t*-test), but more strongly inhibited in the presence of D2 receptor antagonists eticlopride and sulpiride from 36% to 7% (*n* = 32 connections, *p* = 0.005, two-tailed paired samples *t*-test). **e** PI is abolished following bath application of quinpirole but partially recovers following a 30 min wash-out. **f** PI is reduced in the presence of D1 agonist SKF-81297 from 4.2 mV to 2.9 mV (*n* = 22 connections, *p* = 0.006, two-tailed Wilcoxon signed-ranks test) and abolished in the presence of D2 agonist quinpirole from 6.5 mV to 0.3 mV (*n* = 20 connections, *p* = 0.000089, two-tailed Wilcoxon signed-ranks test). **g** Example of PI mediated pause in a postsynaptic ChIN. The presynaptic action potential time is marked with a red dashed line. The pause is blocked following bath application of D2 receptor agonist quinpirole. **h**, **i** Postsynaptic response amplitude in a Striatal ChIN during quinpirole wash-in for light evoked input from TH neurons (red) and feed-forward polysynaptic inhibition (black), or DhβE wash-in on feed-forward inhibition (orange). Input from TH neurons shows gradual attenuation, while polysynaptic inhibition is abruptly gated by either DhβE or quinpirole.

To study the involvement of dopamine in regulating polysynaptic inhibition, we first verified that optogenetic stimulation of dopaminergic terminals indeed evoked dopamine release in the striatum of DAT-Cre mice. We, therefore, measured the striatal dopamine concentration using fast scan cyclic voltammetry in slices from DAT-Cre and VGAT-Cre mice that had been virally transduced in the midbrain to express ChR2. As expected, optogenetic stimulation evoked a robust dopamine response in DAT-Cre mice, but not in VGAT-Cre mice (Fig. 8a, b and Supplementary Fig. 6e). We then repeated the optogenetic pre-pulse protocol in the presence of D1- and D2-type dopamine receptor blockers. Bath application of a D1 receptor antagonist (SCH-23390, 10 µM) did not prevent the attenuation of polysynaptic inhibition (71 ± 6.3% of response amplitude *n* = 33 connections), however, application of D2 receptor antagonists (Eticlopride, 10 µM and Sulpiride, 10 µM) prevented the attenuation (93 ± 6.0% of response amplitude, *n* = 32 connections) suggesting that the observed attenuation following photostimulation is mediated primarily by dopamine acting on D2 receptors (Fig. 8c, d). Lastly, bath application of the D2 agonist quinpirole reversibly suppressed polysynaptic inhibition between ChINs (93.9 ± 2.8% reduction, *n* = 17 connections, Fig. 8e). Application of the D1 receptor agonist SKF-81297 reduced responses to a lesser extent (36 ± 8.2% reduction, *n* = 20 connections), with a large fraction of connections (7/20) not affected at all (Fig. 8f). Moreover, the inhibitory effect of presynaptic ChINs on the discharge of neighboring ChINs was abolished following bath application of quinpirole (Fig. 8g). These results show that while polysynaptic inhibition between striatal ChINs is not mediated by dopaminergic axons, it is strongly suppressed by dopamine acting on D2 receptors. Lastly, we wanted to determine whether the attenuation of polysynaptic inhibition by quinpirole was due to failure of recruiting the mediating interneurons or whether it acted on the GABAergic synapse between THINs and ChINs. To that end, we examined the attenuation of polysynaptic inhibition during gradual bath application of quinpirole. Feed-forward inhibition between ChINs was abruptly eliminated during the course of bath application, suggesting a failure to elicit action potentials in the intermediate neuron, rather than gradual depression of the inhibitory postsynaptic response (Fig. 8h, i). A similar abrupt attenuation was also seen when bath-applying the nicotine receptor antagonist DHβE (Fig. 8h, i), suggesting that in both cases, the underlying cause was insufficient excitation of the intermediate neurons. In contrast, bath application of quinpirole neither blocked the inhibition from THINs onto ChINs nor did it hyperpolarize the membrane potential of THINs (from −67.83 mV to −66.73 mV, *n* = 13 THINs, *p* = 0.20 paired samples *T*-test), further suggesting that the primary source of attenuation of polysynaptic inhibition was a D2 receptor-mediated reduction in acetylcholine release.

## Discussion

**Fig. 9:**
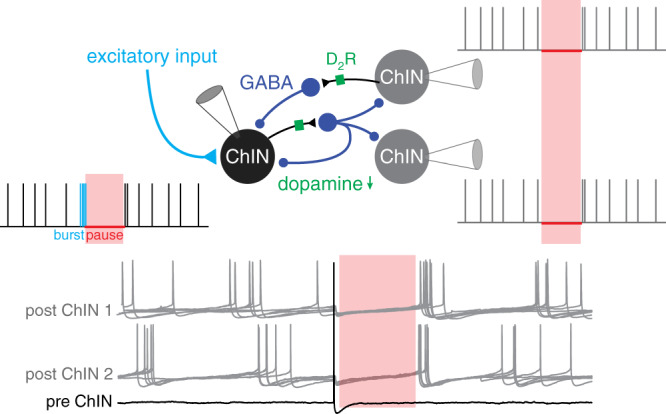
Polysynaptic inhibition between striatal ChINs enhances synchrony and broadcasts the pause signal to neighboring ChINs. Top: following common excitatory input in a subset of ChINs, polysynaptic inhibition can broadcast a synchronous pause in the bursting population (left ChIN) as well as neighboring ChINs that did not burst following the excitatory input (right ChINs). Bottom: presynaptic ChINs can promote synchrony in postsynaptic ChINs by increasing the likelihood of synchronous discharge through rebound spiking after a concurrent inhibition. The scheme illustrates the concurrent discharge following a polysynaptic inhibitory event.

In this study, we show that striatal ChINs are interconnected by a strong and prevalent polysynaptic inhibitory pathway that can affect their firing patterns and promote local synchrony within the ChIN population. We show that synchrony between TANs (putative ChINs) exists in the mouse striatum, as well as reward-related pauses, both of which were modulated by inhibition of striatal GABAergic neurons, thus suggesting a role for polysynaptic inhibition. We identify TH-expressing interneurons as mediators of polysynaptic inhibition and describe two afferent inhibitory inputs to ChINs, originating from GABAergic and dopaminergic midbrain neurons. Neither of these midbrain afferents mediate polysynaptic inhibition of ChINs, however, polysynaptic inhibition and the resulting synchrony are modulated by dopamine via D2 receptors. We, therefore, propose polysynaptic inhibition between ChINs as an intrastriatal mechanism for sculpting the activity patterns of the ChIN population, as well as an additional mode of interaction between the dopaminergic and cholinergic systems in striatal function (Fig. 9).

The inhibitory connection between ChINs is a robust feature of the striatal microcircuitry. It is found in mice and rats^15,35^ and in our hands was seen in all tested mouse lines and a wide range of postnatal ages. The nature of this polysynaptic inhibition is different from other interactions between excitatory neurons such as cortical pyramidal neurons, which exhibit frequency-dependent recruitment of GABAergic interneurons via facilitating excitatory synapses^56,57^. In the case of ChIN polysynaptic inhibition, strong inhibitory responses can be evoked by single presynaptic action potentials; however, responses are attenuated in repetitive stimulation of the presynaptic ChIN, thereby acting as a low-pass filter. Polysynaptic inhibition between ChINs was exceptionally strong, with responses that exceeded even the reported amplitudes of connections between fast-spiking interneurons and MSNs^58–60^. The spatial extent of polysynaptic inhibition between ChINs in our slice recordings was a few hundreds of µm, with the largest recorded distance being 521 µm. This is, however, an underestimate of the interaction range due to cutting of processes in the slice preparation, as well as the constraints of multi-neuron patching in slices. Our data support the involvement of THINs in mediating the polysynaptic pathway (Fig. 5), however, the THIN population is not homogeneous^61^ and it is likely that only specific subtypes form reciprocal synaptic connections with ChINs. Moreover, our data do not exclude the possibility that other GABAergic afferents^31^ or interneuron types that were not studied here may also contribute to the recurrent inhibition between ChINs. Furthermore, modulation of the polysynaptic pathway is observed by somatostatin expressing interneurons, which may gate polysynaptic input by depressing distal dendrites on cholinergic neurons.

As seen in primate striatum^16–18^, TANs (putative ChINs) in mouse striatum were shown to be correlated in their baseline firing, and exhibited synchronous pauses in relation to reward-related events (Fig. 4). Optogenetic inhibition of striatal GABAergic neurons increased the firing rate of TANs while decreasing the degree of correlation between them and shortened the pause events. This suggests that the local GABAergic circuitry in the striatum is involved in shaping the activity patterns of the TAN network, and not only afferent inputs. The effect of optogenetic inhibition on TANs was significant yet not robust. This could be due to insufficient silencing of GABAergic interneurons, especially those that strongly respond to nicotinergic inputs^45,62,63^. Global suppression of striatal inhibitory neurons may also cause opposite effects on selected populations due to differences in viral transfection and complex intrastriatal connectivity patterns^42,44,47,64^.

In primates, correlated activity of TANs was enhanced in dopamine-depleted monkeys^16^, which is in agreement with our findings regarding the attenuating effect of dopamine on polysynaptic inhibition. Synchronized ChIN activity was shown to induce dopamine release^14^ as well as feed-forward inhibition onto striatal projection neurons^15^, suggesting that it is an important feature of their network activity. Such correlations between ChINs can be caused by common afferent inputs. ChINs receive afferent inputs from multiple excitatory and inhibitory sources, both of which can induce spike synchrony across neighboring ChINs by synchronous input. Inhibitory inputs such as those from the midbrain, globus pallidus, and cortex can efficiently synchronize ChINs by rebound spiking, which is further enhanced by specific membrane conductances^65–67^ and delayed excitation^68^. In addition to the afferent common inputs, we propose that correlated activity is also mediated by polysynaptic inhibition between ChINs. We showed that even a single action potential in a presynaptic ChIN can shape the spiking pattern in its neighbors, inducing a synchronized pause-rebound pattern triggered by the polysynaptic inhibition. A similar synchronization mechanism based on polysynaptic inhibition and rebound firing was proposed for hippocampal pyramidal neurons^69^. The synchronous pause of TANs was reported to often, but not always, follow a burst of action potentials^10,11,28,70,71^. The initial burst could induce a pause in spiking ChINs^72^, however, it does not account for the ChINs displaying such a pause without a preceding burst. We showed the existence of both forms of pauses associated with reward-related events in behaving mice. The polysynaptic inhibition between ChINs ensures the broadcasting of pauses to neighboring ChINs even in the absence of burst discharge evoked by excitatory input, thereby acting as a network facilitator of the synchronized pause (Fig. 9). It can also underlie the occurrences of multiple pause/rebound events observed in ChINs under certain conditions^10,16^. The nicotinic receptor desensitization results in frequency-dependent depression^35^ (Fig. 5d) and may require synchronous firing of multiple ChINs to be activated. Interestingly, recovery from desensitization is more likely to occur after longer inter-spike intervals, such as the synchronized pauses, which would also result in larger nicotinic responses from ChINs to their postsynaptic partners. The regulation of ChIN polysynaptic inhibition by dopamine may also explain the occurrence of pauses with different properties upon aversive and appetitive stimuli^5,6^.

Midbrain GABAergic neurons were previously shown to selectively target ChINs in the nucleus accumbens by strong perisomatic inhibition^29^. Here we showed that ChINs in the dorsal striatum also receive such input from midbrain GABAergic afferents, and also appear to provide selective and perisomatic input (Figs. 6, 7 and Supplementary Fig. 4). This suggests that the GABAergic midbrain projection onto ChINs is a general feature of the striatal circuitry, although its respective functional roles in the ventral and dorsal striatum are still unclear. The second GABAergic midbrain projection onto ChINs is provided by the dopaminergic midbrain axons. Co-release of dopamine with GABA and glutamate onto striatal neurons has been reported previously^23,49,68,73,74^. Our data show that ChINs in dorsal striatum receive prominent GABAergic input, which may be different in other striatal regions^23^. Such GABAergic responses and modulation of polysynaptic inhibition were observed also in TH-Cre mice (Supplementary Fig. 6), although it should be noted that these different mice lines vary in the expression patterns of neurotransmitters in a topographic manner^75,76^.

We showed a reduction in polysynaptic inhibition following application of D2 receptor agonists and to a lesser extent by D1 receptor agonists (Fig. 8). Presynaptic attenuation of cholinergic transmission by D2 receptors has been suggested previously^77–84^ and by itself could be sufficient for preventing the recruitment of the intermediate neuron. Our data suggest that the primary cause for the attenuation of ChIN polysynaptic inhibition is indeed the reduction in the strength of the nicotinic synapses driving the intermediate interneurons, and not their intrinsic excitability or the GABAergic input they provide to postsynaptic ChINs. The relationship between dopamine and acetylcholine in the striatum is a complex and dynamic one^3^, with opposite interactions under certain conditions^9^ and synergistic in others^13,14^. Our findings present a novel form of interaction, in which dopamine affects the cholinergic network by modulating the degree of coupling between ChINs. Discharge of midbrain dopaminergic neurons induces dopamine release across large volumes in the striatum, serving as a global dopamine signal. In contrast, ChIN intrinsic coupling can facilitate synchrony among neighboring ChINs that, in turn, regulates local dopamine release in the striatum^85^.

## Methods

### Animals

Experiments were performed using DAT-Cre mice (C57Bl/6J)^50^, TH-Cre mice (TH-Cre [Tg(TH-Cre)12Gsat)^86^, VGAT-Cre mice (C57BL/6J)^51^, SOM-Cre mice (Jackson #018973)^87^, NPY-Cre mice (Jackson #027851)^88^, 5HT3a-Cre (Tg(Htr3a-cre)NO152Gsat/Mmucd)^89^, and wild-type mice (C57Bl/6J). Both male and female mice were used throughout the experiments. Mice were kept on a 12 h day/night cycle and provided with food and water at libitum. Animal rooms were kept between 20 °C and 22 °C and at 50–65% air humidity at all times. All experiments were performed with approval of the local ethical board, Stockholm’s Norra Djurförsöketiska Nämnd and in accordance with the European Communities Council Directive of November 24, 1986 (86/609/EEC). In vivo recordings used wild-type mice and VGAT-IRES-Cre mice and were approved by the University of California, Los Angeles Chancellor’s Animal Research Committee.

The total number of mice used for this study, including for supplementary experiments and auxiliary experiments not listed in the manuscript, is 281. This is divided as follows per transgenic line: DAT-Cre 76, TH-Cre 30, VGAT-Cre 75, SOM-Cre 30, NPY-Cre 6, 5HT3a-Cre 5, wild type 59.

### Slice preparation

Adult mice aged P30 to P140 were anesthetized with isoflurane (VM Pharma AB, Sweden) prior to being decapitated, whereupon brains were extracted while submerged in ice cold cutting solution consisting of (in mM): KCl 2.5, NaH_2_PO_4_ 1.25, CaCl_2_ 0.5, MgCl_2_ 7.5, glucose 10, NaHCO_3_ 25, and sucrose 205. Parasagittal sections of 250 μm thickness were cut using a VT1200S Vibratome (Leica, Japan) at an angle of 10° and subsequently left to recover for 30 min in 35 °C artificial cerebrospinal fluid (ACSF) containing (in mM): NaCl 125, KCl 2.5, MgCl_2_ 1, NaH_2_PO_4_ 1.25, CaCl_2_, Glucose 25, and NaHCO_3_ 25. Slices were maintained at room temperature from recovery until recording at 35 °C. Cutting solution and ACSF were continuously infused with carbogen (95% O_2_, 5% CO_2_) throughout the procedure.

### Patch clamp electrophysiology

Borosilicate glass pipettes were pulled using a P1000 Micropipette Puller (Sutter Instrument, USA) for a resistance of 6–8 MΩ and filled with intracellular solution. For most experiments a high-chloride intracellular solution was used containing (in mM): K-gluconate 105, KCl 30, Na2-Phosphocreatine 10, HEPES 10, ATP-Mg 4, GTP-Na 0.3. Experiments testing the effect of polysynaptic inhibition on spontaneous firing rates used a physiological chloride intracellular solution containing (in mM): K-gluconate 132.5, KCl 2.5, Na2-Phosphocreatine 10, HEPES 10, ATP-Mg 4, GTP-Na 0.3. Cell-attached recordings were performed using pipettes filled with ACSF as described above. For post-hoc staining experiments, 0.1% neurobiotin was added to the intracellular solution. Neurons were selected by Infrared-Differential Interference Contrast (IR-DIC) imaging on a BX51WI (Olympus, Japan) upright microscope using a ×40 long-working-distance immersion objective, controlled through MicroManager 1.4.22 (UCSF, USA). ChINs were identified by their large somata and their identity was confirmed electrophysiologically in whole-cell patch clamp configuration. Wide-field fluorescent imaging was used to localize afferent fibers and in a subset of experiments fluorophore-labeled ChINs. Once a whole-cell patch was achieved, light-evoked responses were recorded at V_H_ = −75 mV in current-clamp on a MultiClamp 700B (Molecular Devices, USA), digitized at 10 KHz on an ITC-18 (HEKA, USA) and acquired with Igor Pro 6.3 (Wavemetrics, USA). Afferent fibers were stimulated through an ocular-mounted blue LED producing 6.4 mW light under the objective, controlled through a SLA-1200-2 LED driver (Mightex, USA). Baseline responses were recorded without receptor antagonists present in the ACSF. For extracellular stimulation, an Iso-Flex stimulus isolator (A.M.P.I., Israel) produced a current pulse through a large-diameter glass pipette tuned to reliably evoke synaptic inhibition from a distance of approximately 150 μm to the nearest patched neuron. In nicotine puffing experiments, a patch pipette was loaded with 100 μM nicotine in ACSF and aimed at the somata of patched neurons from approximately 50 μm distance. Positive pressure was applied for 250 ms through a Picospritzer III (Parker Hannifin, USA) at 1 min intervals.

### Chemicals used in ex vivo experiments

Unless otherwise indicated, compounds were bath applied through the slice perfusion system and continuously perfused during patch clamp recordings under the stated condition. A complete list of chemicals used is provided in Supplementary Table 1.

### Electrophysiological response analysis

Evoked synaptic responses were averaged per cell over >10 alternating repetitions in Igor Pro 6.34A (Wavemetrics, USA). Response area was determined by subtracting a baseline (10 ms prior to event onset) from the response for a maximum of 100 ms or until the response dropped below baseline. For peak amplitude analysis, the maximum deviation from baseline was used. In the case of feedback inhibition, the response to the second pulse stimulus was baseline-corrected and subsequently subtracted from the first pulse, as this subsequent response does not contain nicotine-dependent polysynaptic input (Supplementary Fig. 1d). Statistical analysis was subsequently performed using SPSS Statistics 17.0 (IBM, USA) and Origin Pro 9.0 (OriginLab Corporation, USA).

### Combined chemogenetic inhibition and optogenetic excitation

For experiments where we combined chemogenetic inhibition with optogenetic excitation (local striatal interneurons, midbrain afferents), the protocol was as follows: 1. Cholinergic neurons were recorded in whole-cell patch clamp configuration and alternatingly tested for polysynaptic inhibition and light-induced input at 15 s intervals as described above for at least 10 repetitions. 2. If a polysynaptic response and a light-evoked response was evident, 10 μM clozapine-N-oxide (CNO) in ACSF was perfused in the bath for at least 5 min. 3. Following bath application of CNO, cholinergic neurons were again alternatingly tested for polysynaptic input and light-induced input. Responses were subsequently averaged and analyzed post-hoc as above (Fig. 5b and Supplementary Fig. 1d). To assess the degree of light-induced synaptic depletion, following each trial the light stimulation was repeated but instead of polysynaptic inhibition, an extra light pulse was given in its stead to compare the initial light response with the light response that occurred at the normal time of polysynaptic inhibition. Light-induced synaptic depletion varied between inputs (Supplementary Fig. 4i) and interference is, therefore, taken as indicative but not conclusive evidence of a population’s involvement in polysynaptic inhibition.

### In vivo electrophysiology during Pavlovian reward conditioning

All animal procedures for in-vivo electrophysiology were approved by the University of California, Los Angeles Chancellor’s Animal Research Committee. Experiments in Fig. 4 involved male C57Bl/6J mice. Animals underwent a first surgical procedure under aseptic conditions and isoflurane anesthesia on a stereotaxic apparatus. Every surgical procedure involved attaching stainless steel head fixation bars on the skull. Additionally, for experiments involving optogenetic inhibition of GABAergic neurons (Fig. 5), 500 nL of AAV5/EF1a-DIO-eNpHR3.0-mcherry was bilaterally injected in the striatum (coordinates relative to bregma: 1.0 mm anterior, 2.2 mm lateral, 3.3 mm ventral) of male VGAT-IRES-Cre mice (The Jackson Laboratory, stock no. 028862) using pulled glass pipettes (Nanoject III, Drummond Scientific). AAV was obtained from the University of North Carolina Vector Core. All animals were individually housed after surgery, and recovered for at least 2 wks (3 wks for optogenetics experiments) before beginning behavioral conditioning. For conditioning, mice were food restricted to maintain their weight at around 90% of their baseline level, and given water ad libitum. Animals were initially habituated to the head fixation apparatus and to reliably consume uncued rewards (5 μL, 10% sweetened condensed milk), which were delivered via actuation of an audible solenoid valve. The reward delivery and lick meter port was located ~5 mm directly in front of the mouth, and animals had to extend their tongue out of the mouth to register as a lick. Subsequently, animals were trained on a Pavlovian reinforcement task using an olfactory cue, consisting of isoamyl acetate diluted 1:10 in mineral oil, and diluted another factor of 10 by mixing with clean air in an olfactometer (total air flow was 1.5 L min^−1^). Behavioral trials consisted of a 1 s odor cue, followed by a reward 3 s after cue onset (100 trials per session, 25 ± 5 s intertrial interval). Anticipatory licking was defined as a bout of licking that began between 0 to 3 s after cue onset. Animals were trained for 3–5 days before undergoing electrophysiological recordings. For recordings, a second surgery under isoflurane anesthesia was completed 6-12 h prior to recording to create a rectangular craniotomy above the striatum. The dura was removed to facilitate device insertion. An additional craniotomy was made over the posterior cerebellum to accommodate a silver/silver-chloride electrical reference wire. After inserting a 256 electrode silicon microprobe (4 prongs, 64 electrodes per prong), mineral oil was placed in the craniotomy, and recording commenced after 40 min. For optogenetics experiments, the silicon microprobe was attached to a pair of optical fibers (Thor Labs, 0.2 mm diameter, 0.22 NA)1. The device was inserted in the coronal plane, and the target coordinates relative to bregma were 1.0 mm anterior, 1.9 to 2.5 mm lateral, and 4.0 to 4.2 mm ventral. A 300 s resting period was recorded followed by 100–150 behavioral trials, and for the optogenetics experiments half the trials were randomly paired with a laser stimulus (532 nm, 8 mW output per fiber, 8 s continuous duration starting 2 s before cue onset). In some animals used for optogenetics experiments, recordings were carried out in each hemisphere on successive days. Recordings were performed at a sampling rate of 25 kHz per electrode with a commercial data acquisition system (Intan Technologies), and spike sorting was carried out with custom Matlab scripts or open-source software (Kilosort)^38^.

### Analysis of in vivo recordings

For classification of different cell types^40^, putative FSIs were defined by a narrow spike waveform (maximum width = 0.475 ms), and relatively high baseline firing (minimum rate = 0.25 Hz). MSNs and TANs were both defined by wider waveforms (minimum width = 0.55 ms, maximum width = 1.25 ms). TANs were separated from MSNs by the regularity of their baseline firing (maximum coefficient of variation = 1.5). The minimum baseline firing was defined as 0.02 Hz for MSNs and 2 Hz for TANs, and the maximum was defined as 10 Hz for both cell types. All analysis of firing rate responses after rewards used trials with anticipatory licking. Firing rate was binned in 5 ms time steps and smoothed with a Gaussian convolution filter (SD = 25 ms). The *z*-scored firing rate was obtained with respect to a 1 s baseline period immediately preceding cue presentation. A cell was identified as burst-pause if its firing rate exceeded 1 SD above baseline for at least one time step from 0 to 100 ms of reward delivery, and was less than 1 SD below baseline for at least one time step from 100 to 250 ms of reward delivery. A cell was identified as only pause if its firing rate did not exceed 1 SD above baseline for any time steps from 0 to 100 ms of reward delivery, and was less than 1 SD below baseline for at least one time step from 100 to 250 ms of reward delivery. A rebound burst after the pause was defined as firing rate exceeding 1 SD for at least one time step from 500 to 1000 ms after reward delivery. The pause duration was estimated from the time span between the −0.5 SD firing rate crossing.

### Cross-correlation analysis

Spike time cross-correlograms (CCGs) were calculated in time steps of 5 ms. The burst-pause and only pause TAN populations were pooled to increase the number of simultaneously recorded cell pairs. The resting CCG was calculated from the first 300 s of the recording during which no stimuli or rewards were presented. The cued licking CCG was calculated by concatenating spike times from the first 50 cue-reward trials with anticipatory licking (spike time range per trial: −3 to 3 s from reward). This ensured that both the resting and cued licking CCG contained spike time data from an equivalent total time span (300 s). For recordings involving optogenetic inhibition of GABAergic neurons, the CCG was calculated by concatenating spike times from the first 36 cue-reward trials with anticipatory licking and either without laser (Laser Off) or with laser (Laser On). The spike time range per trial was again −3 to 3 s from reward. This ensured that both the Laser Off and Laser On CCGs contained spike time data from an equivalent total time span (216 s). The cross-correlation modulation index, η, was calculated by the expression:

η = (S_center − S_baseline)/(S_center + S_baseline)

where S_center is the mean spike count within ±20 ms of zero time lag of the CCG, and S_baseline is the mean spike count in the outer 100 ms of the CCG.

### Carbon fiber microelectrodes

Carbon fiber electrodes were fabricated by aspirating 7 μm diameter carbon fibers (Cytec engineered materials, USA) into borosilicate glass capillaries (1.2 mm O.D., 0.69 mm I.D., Sutter Instrument, USA). The capillaries were subsequently pulled with a P97 micropipette puller (Sutter Instrument, USA) and sealed with epoxy (EpoTek 301, Epoxy Technology, USA). The electrode tips were polished at a 45° angle on a diamond dust-embedded micropipette-bevelling wheel (Model BV-10, Sutter Instrument, USA). Electrodes were tested by bath applying known concentrations of dopamine. Only electrodes showing good reaction kinetics (as examined in current vs time plots, and current vs voltage plots) were used.

### Fast-scan cyclic voltammetry

A Dagan Chem-Clamp potentiostat (Dagan Corporation, USA) and two data acquisition boards (PCI-6221, National Instruments, USA) run by the TH 1.0 CV program (ESA, USA) were used to collect all electrochemical data. Cyclic voltammograms were obtained by applying a triangular waveform potential (−0.4 to +1.3 V vs Ag/AgCl) repeated every 100 ms at a scan rate of 200 V s^−1^ (low pass Bessel filter at 3 kHz). Each cyclic voltammogram was a background-subtracted average of ten successive cyclic voltammograms taken at the maximum oxidation peak current. All electrodes were allowed to cycle for at least 15 min prior to recording to stabilize the background current. The recorded current response was converted to dopamine concentration via in vitro electrode calibration with standard dopamine solution prior to each experiment. Acquired data were analyzed and plotted using Matlab 2015A (Mathworks, USA) routines and statistical analysis was performed using Prism 6.0 (GraphPad Software, USA).

### Histology

Following recording, slices containing neurobiotin-filled neurons were fixed overnight in Lana’s fix. Slices were subsequently washed four times in 0.01 M PBS, then placed in 0.5 μl streptavidin Cy5 (Thermo Scientific, USA) in 500 μl 0.1 M PB containing 0.3% Triton X-100 overnight. For TH-staining, slices were incubated in a 1:1000 Anti-Tyrosine Hydroxilase antibody (AB152, lot 3114503, Millipore Sigma, USA). Slices were washed again before mounting on Superfrost Plus microscope slides (Thermo Scientific, USA) using Fluoromount Aqueous mounting medium (Sigma, USA). Mounted tissue images were subsequently acquired using the Zen 2 black edition software (Carl Zeiss AG, Germany).

### Viral transduction for ex vivo experiments

Mice were anesthetized using isoflurane and head-fixed in a stereotaxic frame (Kopf, USA). Viral suspensions were loaded in a 10 µL Nanofil syringe equipped with a 34G beveled needle (Hamilton, USA). For midbrain injections, 1 µL of viral suspension was injected at (in mm from bregma): mediolateral 0.5, dorsoventral 4.7, anteroposterior -(3 * X) / 4.21 where X denotes the distance between lambda and bregma, targeting the ventral tegmental area and surrounding tissue. Injections targeting the SNc were performed at: mediolateral 1.1, dorsoventral 4.2, anteroposterior -(3.1 * X) / 4.21. Most midbrain injections, including those for TeLC and dtA lesion experiments, targeted the above VTA coordinates, with a few control experiments in which the SNc coordinates were used. The response to optogenetic stimulation was not different between cases as well as in striatal injections with the retrograde AAV injections, therefore, data were pooled. Striatal injections were made at: mediolateral 1.6, dorsoventral 3.0 & 2.8, anteroposterior 0.8 in mm relative to bregma. For these injections, 0.5 µL of viral suspension was injected at each depth, both for AAV5 and AAVrg serotypes. In all cases viral suspensions were injected at 0.1 µL min^−1^. For combined vectors (e.g., both ChR2 and hM4D(Gi)) both viral suspensions were mixed prior to loading and injected simultaneously. Post-injection, the canula was left in place for at least 5 min before changing position or retracting. Following surgery, mice received an IP injection of the buprenorphine based analgesic Temgesic (Reckitt Benckiser, Switzerland) and were allowed to recover for at least two weeks before recording for Striatal injections, and at least four weeks for midbrain injections.

### Viral constructs

Various viral constructs were used throughout this study. A complete list of constructs, titer and source is provided in Supplementary Table 2.

### 6-OHDA lesioning

A total of 9 mice (male and female) were anesthetized using isoflurane and head-fixed in a stereotaxic frame (Kopf, USA). Each mouse was injected unilaterally with 1 µl 6-OHDA-HCl (3.75 µg µl^−1^ in 0.02% ascorbic acid) in the medial forebrain bundle at (in mm): −1.2 antero-posterior, +1.2 medio-lateral and −4.8 dorso-ventral. Following surgery, mice received Temgesic (0.1 mg Kg^−1^, Reckitt Benckiser, England) and were allowed to recover for at least 3 weeks. All mice were confirmed to display parkinsonian-like rotation behavior typical of successful lesions^90^.

### Reporting summary

Further information on research design is available in the Nature Research Reporting Summary linked to this article.

## Supplementary information

Supplementary Information

Reporting Summary

## Data Availability

The data that support the findings of this study are available from the authors on reasonable request, see author contributions for specific data sets. Source data are provided with this paper.

## Source data

Source data
