# Supplementary material for: Polysynaptic inhibition between striatal cholinergic interneurons shapes their network activity patterns in a dopamine-dependent manner
Source: Nat Commun. 2020 Oct 9;11:5113. doi: 10.1038/s41467-020-18882-y (PMC7547109; doi:10.1038/s41467-020-18882-y)
Supplement: Supplementary file 1 — Supplementary Information [file 41467_2020_18882_MOESM1_ESM.pdf]

Supplementary Table 1. Chemicals used in ex vivo electrophysiology experiments

Table 1: chemicals used in ex vivo electrophysiology experiments			
**compound**	**chemical name**	**concentration**	**source**
NBQX	2,3-dioxo-6-nitro-7-sulfamoyl-benzo[f]quinoxaline	10 µM	Tocris
D-AP5	D-(-)-2-Amino-5-phosphonopentanoic acid	50 µM	Tocris
GABAzine	6-Imino-3-(4-methoxyphenyl)-1(6H)-pyridazinebutanoic acid hydrobromide	10 µM	Tocris
DHβE	(2S,13bS)-2-Methoxy-2,3,5,6,8,9,10,13-octahydro-1H,12H-benzo[i]pyrano[3,4-g]indolizin-12-one hydrobromide	1 µM	Tocris
SCH-23390	R(+)-7-Chloro-8-hydroxy-3-methyl-1-phenyl-2,3,4,5-tetrahydro-1H-3-benzazepine hydrochloride	10 µM	Sigma-Aldrich
Eticlopride	3-Chloro-5-ethyl-N-[[(2S)-1-ethyl-2-pyrrolidinyl)methyl]-6-hydroxy-2-methoxy-benzamide hydrochloride	10 µM	Tocris
Sulpiride	(S-)-5-Aminosulfonyl-N-[(1-ethyl-2-pyrrolidinyl)methyl]-2-methoxybenzamide	1 µM	Tocris
SKF-81297	(±)-6-Chloro-2,3,4,5-tetrahydro-1-phenyl-1H-3-benzazepine hydrobromide	10 µM	Tocris
Quinpirole	(4aR-trans)-4,4a,5,6,7,8,8a,9-Octahydro-5-propyl-1H-pyrazolo[3,4-g]quinoline hydrochloride	10 µM	Tocris
TTX	Octahydro-12-(hydroxymethyl)-2-imino-5,9:7,10a-dimethano-10aH-[1,3]dioxocino[6,5-d]pyrimidine-4,7,10,11,12-pentol	0.5 µM	Tocris
4-AP	4-Aminopyridine	100 µM	Tocris
CNO	8-Chloro-11-(4-methyl-4-oxido-1-piperazinyl)-5H-dibenzo[b,e][1,4]diazepine	10 µM	Tocris
Nicotine[1]	(S)-(-)-1-Methyl-2-(3-pyridyl)pyrrolidine (+)-ditartrate salt	100 µM	Tocris

[1] nicotine was applied topically trough a patch pipette.

Supplementary Table 2. Viral constructs used in this study.

Table 2: viral constructs			
**Name**	**type**	**Titer**	**Source**
pAAV-EF1a-double floxed-hChR2(H134R)-mCherry-WPRE-HGHpA	AAV5	5.3e12 GC/ml	Addgene, #20297
pAAVrg-EF1a-double floxed-hChR2(H134R)-mCherry-WPRE-HGHpA	AAVrg	1.4e13 GC ml^{-1}	Addgene, #20297
pAAV-hSyn-DIO-hM4D(Gi)-mCherry	AAV5	1.1e13 GC ml^{-1}	Addgene, #44362
pAAV-EF1a-double floxed-hChR2(H134R)-EYFP-WPRE-HGHpA	AAV5	7.7e12 GC ml^{-1}	Addgene, #20298
rAAV5-EF1-Lox-Cherry-lox(dtA)-lox2.ape	AAV5	6.0e12 GC ml^{-1}	Addgene, #58536
AAV5-EF1a-DIO-eNpHR3.0-mcherry	AAV5	3.3e13 GC ml^{-1}	UNC Vector Core
pAAV-hSyn-FLEX-TeLC-P2A-EYFP-WPRE	AAV5	6.4e12 GC ml^{-1}	Wulff laboratory[89]

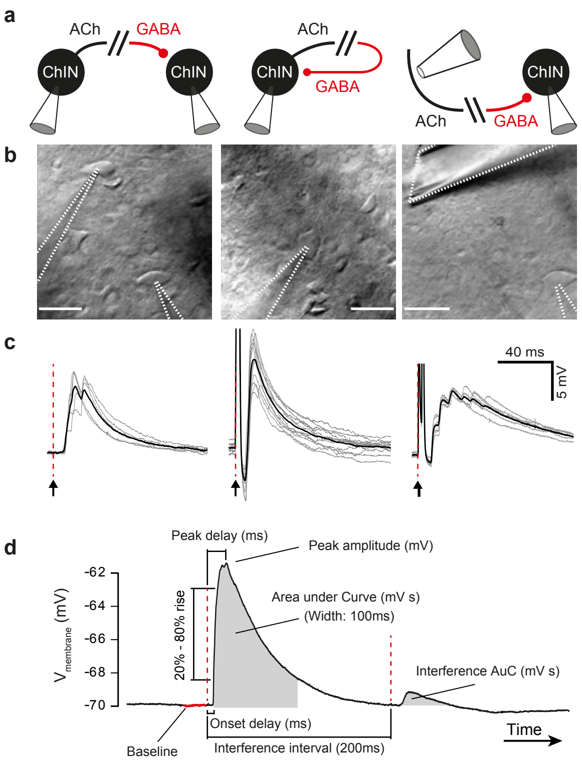

Dorst et al, Supplementary Fig. 1

Supplementary Fig. 1. PI recording configurations. a, Polysynaptic responses were recorded from pairs, triplets, and quadruples of ChINs expressing feed-forward inhibition (left, n = 212 connections), single ChINs expressing feed-back inhibition (middle, n = 315 connections) or ChINs receiving feed-forward inhibition elicited by extracellular stimulation (right, n = 93 connections). **b,** Example DIC images of recording configurations described in a. Scale bar: 30 µm. **c,** Examples of polysynaptic responses recorded in the corresponding configurations. **d,** Response analysis overview. Since PI responses occasionally varied not only in amplitude but also in whether they expressed multiple concurrent inputs, we calculated the integral (area under the curve) of the response as well as peak amplitude. The PI response was calculated as deviation from baseline (average membrane potential during 10 ms prior to stimulus onset) from 10 ms post stimulus onset to 100 ms following stimulus onset or until the response decreased below baseline, whichever came first. Peak amplitude was determined as the maximum deviation from baseline in the 50 ms following stimulus onset and peak delay as the time from stimulus onset to peak.

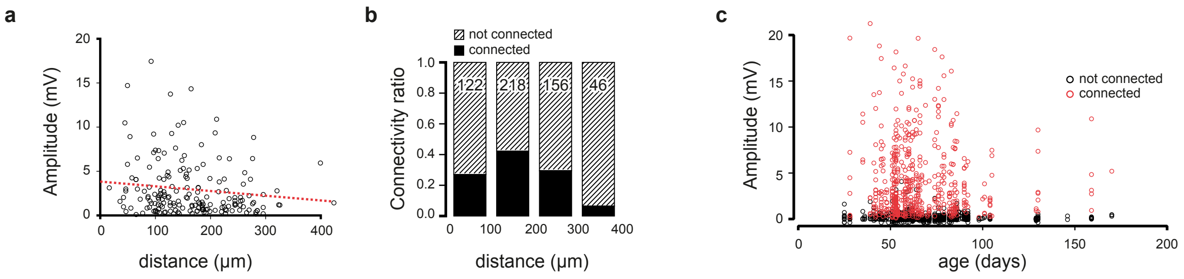

Dorst et al, Supplementary Fig. 2

Supplementary Fig. 2. Response prevalence over distance. a, amplitudes of feed-forward polysynaptic responses only weakly decreased with distance as 4.1 mV - 6.1 µV μm^{-1} (two-sided linear regression, n = 176 feed-forward connections, p = 0.048, F = 4.151, R^2 = 0.023). **b,** the rate of connectivity was significantly associated with distance (Pearson $X^2(3)$ = 36.083, p = 7.2E-8). Numbers in bars indicate tested connections per distance group. **c,** the rate and amplitude of polysynaptic inhibition was not affected by age.

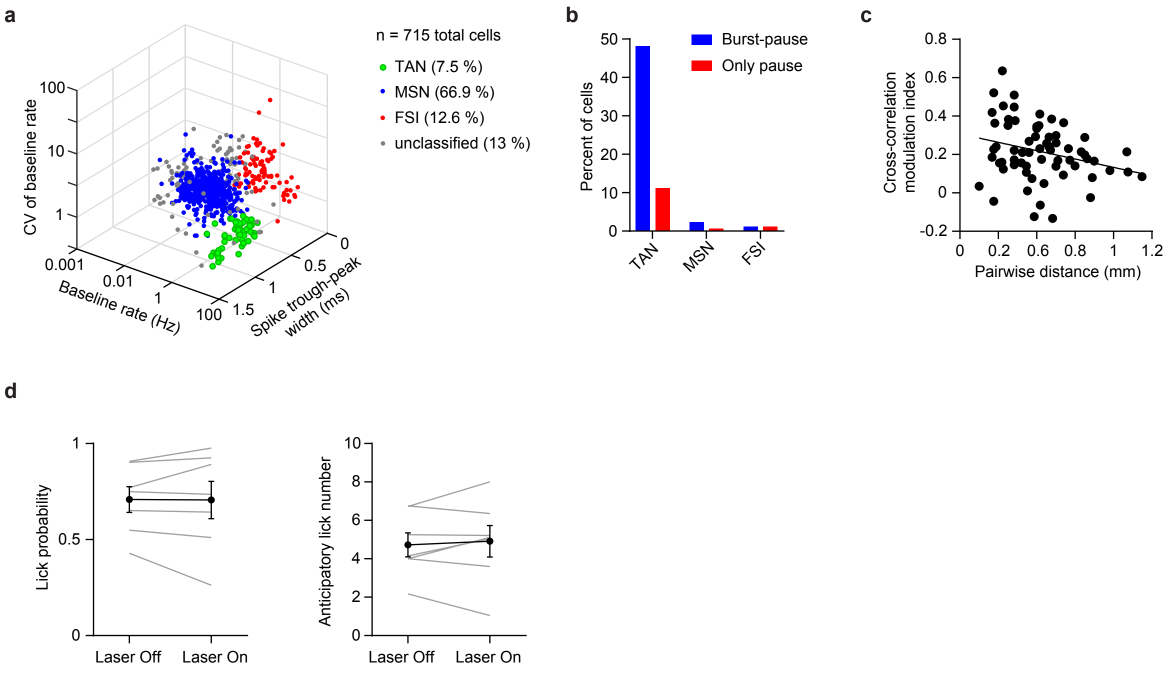

Dorst et al, Supplementary Fig. 3

Supplementary Fig. 3. TAN classification and reward pause properties. a, 3D plot of baseline firing rate as a function of spike trough-to-peak width and coefficient of variation (CV) of the baseline firing for all 715 striatal units recorded from 10 C57Bl/6J mice. Each point is color coded according to the cell's classification. Total number of TANs = 54; MSNs = 478; FSIs = 90. **b**, Percent of reward burst-pause and pause-only cells in each cell type (n = 54 TANs, 478 MSNs, 90 FSIs). **c**, Cross-correlation modulation index as a function of pairwise TAN distance. Data represent cross-correlations from the entire recording session. The modulation index is negatively correlated with distance (n = 65 pausing TAN pairs, Pearson r = -0.27, p = 0.03). The line shows the best line fit. **d**, Behavioral performance in recording sessions with optogenetic inhibition of striatal VGAT-positive neurons. Left: anticipatory licking probability was not significantly altered on trials with laser (n = 7 recording sessions, two-sided paired t-test, p = 0.95. Right: the number of anticipatory licks was not significantly altered on trials with laser (n = 7 recording sessions, two-sided paired t-test, p = 0.6). All data represent mean ± SEM.

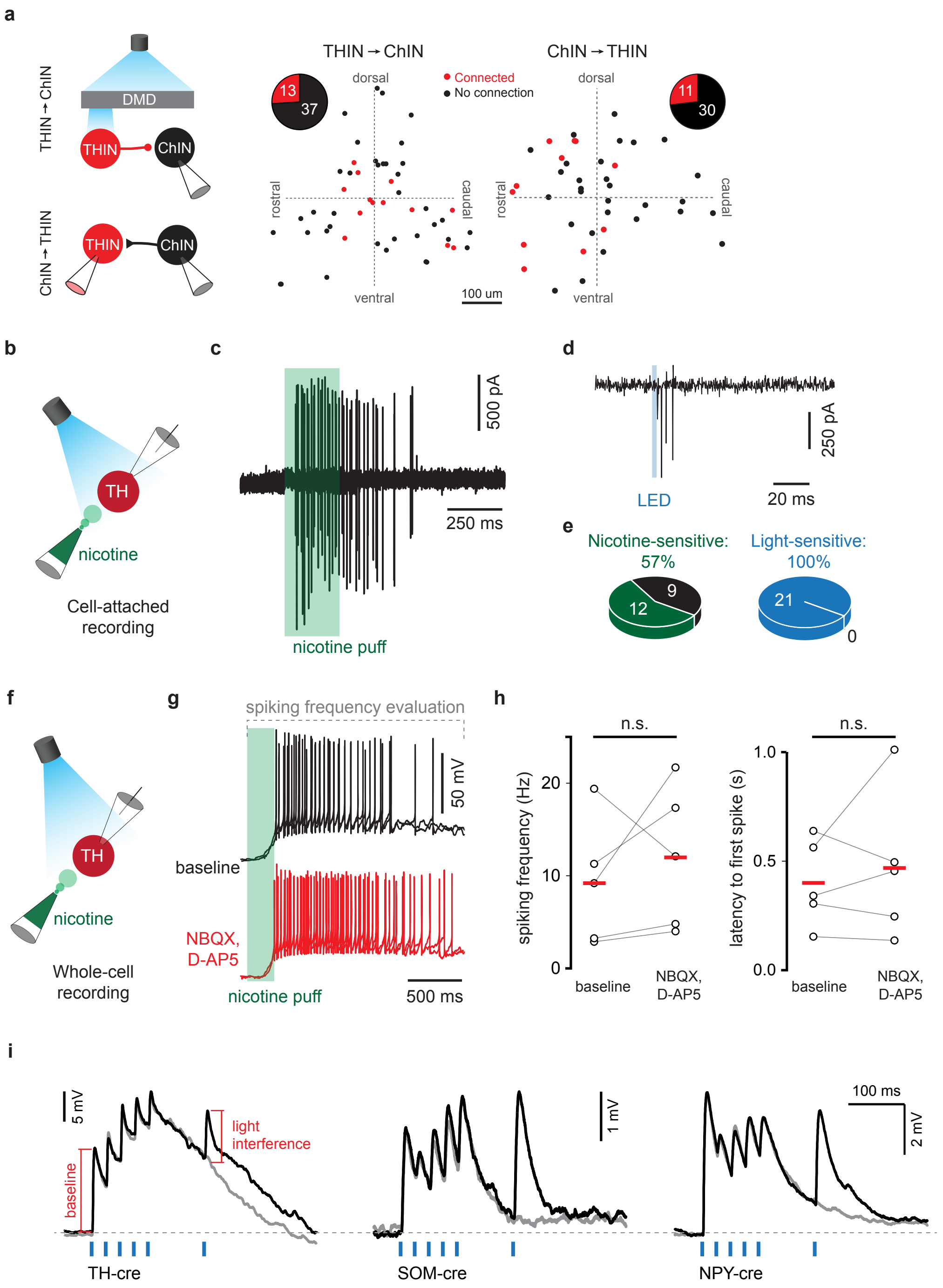

Dorst et al, Supplementary Fig. 4

Supplementary Fig. 4. Striatal THINs express robust reciprocal connections with nearby ChINs. a, synaptic input was observed in 26% of ChINs following structured-light activation of presynaptic TH neurons using a Polygon 400 (Mightex, USA) digital micromirror device (DMD). Mean response amplitude was 0.54±0.06 mV (middle). Right: in paired recordings between ChINs and postsynaptic TH neurons recorded with Cs-based ICS, nicotinic responses were detected in 27% of TH neurons with mean amplitude 0.87±0.17mV. No directional preference was observed for TH to ChIN connections; for ChIN to TH connections, rostral connections were more likely ($p = 0.003$, two-sided Mann-Whitney test). **b,c,** THINs were recorded in cell-attached configuration to better match physiological conditions and nicotine was puffed for 250 ms from a patch pipette positioned nearby. Action potentials were detected in 57% (12/21) tested THINs. **d,e,** 5 ms light pulses were used to verify that THINs recorded in cell-attached mode could fire detectable action potentials. All recorded THINs reliable fired on light stimulation. **f,g,h,** Glutamate receptor antagonists were applied to nicotine-sensitive THINs in whole-cell configuration. This had no effect on firing rate or onset latency ($n = 5$ THINs, $Z = -0.944$, $p = 0.345$ and $Z = -0.135$, $p = 0.893$ respectively, two-sided Wilcoxon Signed Ranks Test). **i,** to assess expected synaptic modulation, we recorded post-synaptic responses in striatal ChINs following light activation of striatal interneurons (blue marks indicate light activation). The initial light evoked response was compared to the response elicited following light interference, when normally the polysynaptic pathway would be activated (black traces). This last light interference pulse was omitted in alternate trials (grey traces) to separate compound responses. Examples are shown for activation of striatal interneurons expressing TH, SOM and NPY. We observed synaptic depression, facilitation and cases with no discernible synaptic plasticity.

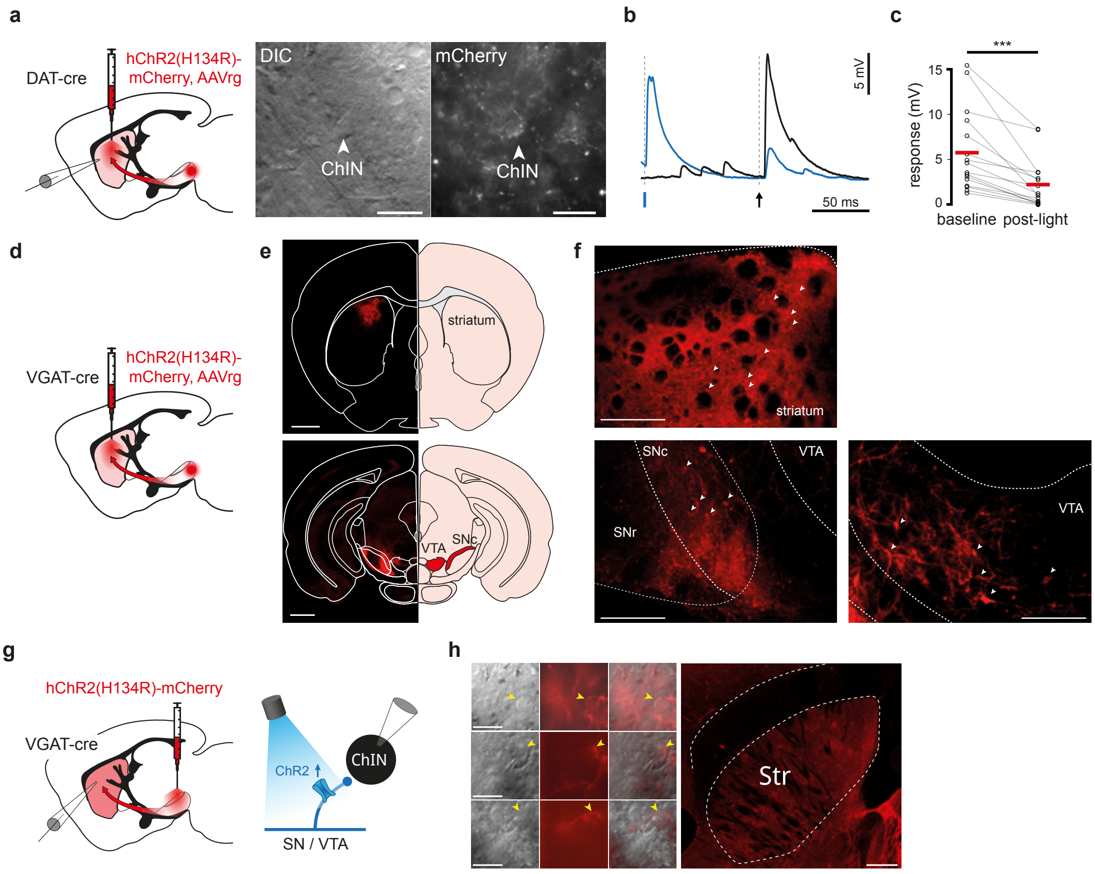

Dorst et al, Supplementary Fig. 5

Supplementary Fig. 5. Midbrain neurons target striatal ChINs, but do not mediate polysynaptic inhibition. a, DAT-cre mice were injected in the striatum with a retrograde-AAV inducing expression of hChR2(H134R)-mCherry in midbrain dopamine neurons (n = 5 mice). Scale bars: 30 µm. **b,** light activation reliable evoked postsynaptic responses in striatal ChINs and **c,** significantly reduced polysynaptic inhibition (n = 15 polysynaptic connections, Z = -3.408, p = 0.00066, two-sided Wilcoxon Signed Ranks Test). **d,** a cre-dependent retrograde-AAV virus injected in the dorsal Striatum of VGAT-cre mice labels gabaergic inputs (n = 3 mice). **e,** the injection site (top) and retrogradely labelled inputs to dorsal striatum (bottom) in coronal sections. Scale bars: 1 mm. **f,** transduced somata (examples marked with arrows) are found near the injection site (top), and in the VTA and SNc (bottom). Scale bars: 200 µm. **g,** striatal ChINs were recorded in VGAT-cre mice injected with an AAV5 expressing hChR2(H134R)-mCherry in midbrain gabaergic neurons (n = 19 mice). **h,** midbrain afferents wrap around ChIN somata in a VGAT-cre mouse. Scale bars (left): 30 µm, (right): 500 µm.

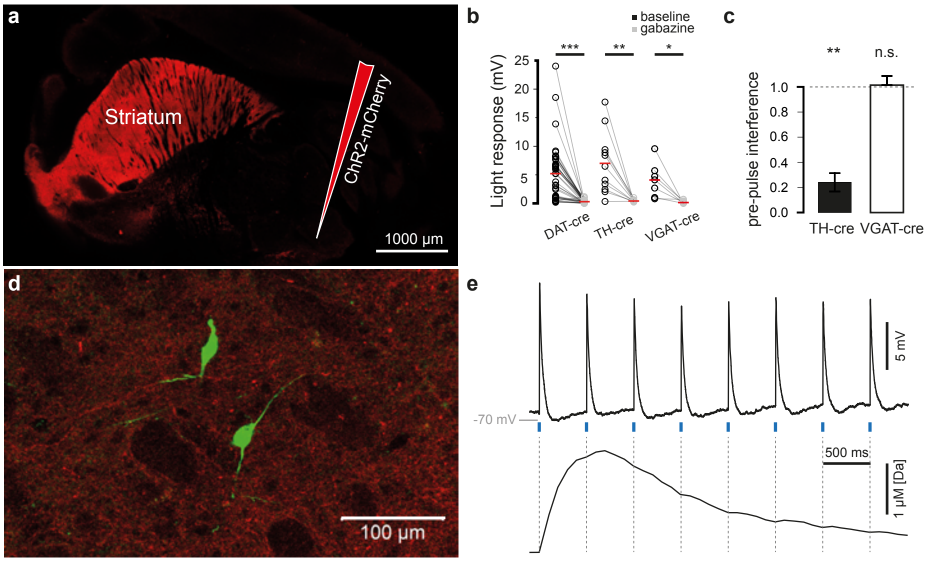

Dorst et al, Supplementary Fig. 6

Supplementary Fig. 6. Midbrain and striatal cells in DAT-cre and TH-cre mice enable light evoked interference in PI and provide gabaergic inhibition of ChINs. a, Parasagittal slice in a DAT-cre mouse transduced with ChR2-mCherry in the midbrain (n = 62 DAT-cre mice were injected with hChR2(H134R) in this manner). **b,** Light-evoked responses were recorded in ChINs from DAT-cre, VGAT-cre, and TH-cre mice transduced with hChR2(H134R) in the midbrain. In all cases responses were abolished by bath application of gabazine (n = 20 responding ChINs, p = 0.000062 DAT-cre; n = 7 responding ChINs, p = 0.019 VGAT-cre; n = 11 responding ChINs, p = 0.002329 TH-cre; two-sided paired samples T-test). **c,** TH-cre animals transduced with hChR2(H134R) show robust attenuation of polysynaptic responses following an optogenetic pre-pulse (response ratio = 0.24, n = 12 connections, Z = -3.059, p = 0.002, 2-tailed Wilcoxon signed ranks test) as opposed to VGAT-cre animals (response ratio = 0.98, n = 35 connections, Z = -0.75, p = 0.45, two-sided Wilcoxon signed ranks test). Error bars represent mean ± SEM. **d,** DAT-cre terminals in the Striatum (red) surround neurobiotin-labelled ChINs (green) (observed in n = 62 DAT-cre mice transduced with hChR2(H134R)). **e,** Light-evoked gabaergic responses in a DAT-cre mouse at 2Hz show little depression in synaptic response (top) but slower onset and faster depletion in FSCV recorded [Da] response (bottom).

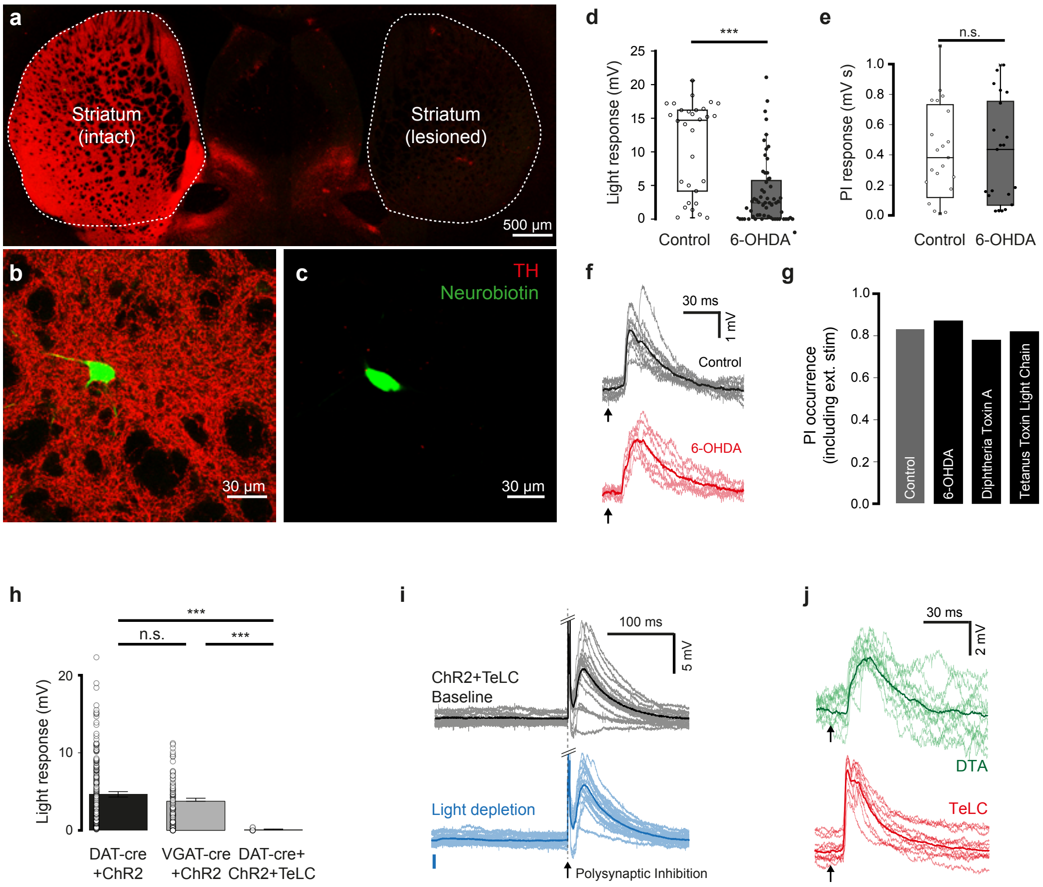

Dorst et al, Supplementary Fig. 7

Supplementary Fig. 7. Lesioning of midbrain dopaminergic afferents does not affect disynaptic inhibition. a-c, in mice lesioned through 6-OHDA injections in the medial forebrain bundle, TH immunoreactivity in the lesioned hemisphere is strongly reduced (n = 4 mice). **d,** in DAT-cre mice transduced with hChR2(h134R)-mCherry in the midbrain and subsequently lesioned with 6-OHDA, the majority of ChINs receive no light-induced input, and light responses are significantly reduced compared to the unlesioned hemisphere (n = 30 unlesioned, 60 lesioned ChINs, p = 0.000007, two-sided t-Test). Boxplots indicate minimum, first quartile, median, third quartile, and maximum. **e-f,** polysynaptic responses were unaffected in the lesioned versus unlesioned hemisphere. Boxplots indicate minimum, first quartile, median, third quartile, and maximum. (n = 21 polysynaptic connections in control and 6-OHDA groups, p = 0.89, two-sided t-Test). **g,** polysynaptic responses could be elicited with the same frequency in control animals versus animals lesioned with 6-OHDA, Diphtheria Toxin A or Tetanus Toxin Light Chain. **h,** light-evoked responses in striatal ChINs in DAT-cre and VGAT-cre animals transduced in the midbrain with hChR2 were of comparable strength (p = 0.228, Z = -1.206, n = 242 postsynaptic ChINs in DAT-cre and 106 ChINs in VGAT-cre animals), but were strongly reduced in DAT-cre animals lesioned with Tetanus Toxin Light Chain compared to unlesioned DAT-cre animals (p = 0.000023, Z = -4.567, n = 7 ChINs in DAT-cre + TeLC animals) and VGAT-cre animals (p = 0.000061, Z = -4.010, two-tailed Mann-Whitney U Test for all comparisons). Error bars represent mean ± SEM. **i,j,** feedback and feed-forward polysynaptic responses exhibited no qualitative changes in DAT-cre mice selectively lesioned with TeLC or DTA, but lacked light-induced interference originating from dopaminergic neurons.
